# Nanograting-Based Dynamic Structural Colors Using Heterogeneous Materials

**DOI:** 10.1007/s40820-024-01554-7

**Published:** 2024-11-11

**Authors:** Jingang Wang, Haibo Yu, Jianchen Zheng, Yuzhao Zhang, Hongji Guo, Ye Qiu, Xiaoduo Wang, Yongliang Yang, Lianqing Liu

**Affiliations:** 1 State Key Laboratory of Robotics, Shenyang Institute of Automation, Chinese Academy of Sciences, Shenyang, 110016 People’s Republic of China; 2 University of Chinese Academy of Sciences, Beijing, 100049 People’s Republic of China

**Keywords:** Dynamic structural colors, Four-dimensional printing, pH-responsive, Nanogrid, Heterogeneous materials

## Abstract

Dynamic structural colors can change in response to different environmental stimuli. This ability remains effective even when the size of the species responsible for the structural color is reduced to a few micrometers, providing a promising sensing mechanism for solving microenvironmental sensing problems in micro-robotics and microfluidics. However, the lack of dynamic structural colors that can encode rapidly, easily integrate, and accurately reflect changes in physical quantities hinders their use in microscale sensing applications. Herein, we present a 2.5-dimensional dynamic structural color based on nanogratings of heterogeneous materials, which were obtained by interweaving a pH-responsive hydrogel with an IP-L photoresist. Transverse gratings printed with pH-responsive hydrogels elongated the period of longitudinal grating in the swollen state, resulting in pH-tuned structural colors at a 45° incidence. Moreover, the patterned encoding and array printing of dynamic structural colors were achieved using grayscale stripe images to accurately encode the periods and heights of the nanogrid structures. Overall, dynamic structural color networks exhibit promising potential for applications in information encryption and in situ sensing for microfluidic chips.

**Supplementary Information:**

The online version contains supplementary material available at 10.1007/s40820-024-01554-7.

## Highlights

- A 2.5-dimensional dynamic structural color based on nanogratings of heterogeneous materials was proposed by interweaving a pH-responsive hydrogel with IP-L photoresist.
- The nanogrid structures exhibit brilliant tuneable structural color, high sensitivity, and ultrafast recovery speeds in response to pH.
- The 4D printing-based grayscale design approach was proposed for the patterned encoding and array printing of dynamic structural colors, promoting their application in patterned printing, information encryption, and microfluidic chip sensing.

**Supplementary Information:**

The online version contains supplementary material available at 10.1007/s40820-024-01554-7.

## Graphical abstract

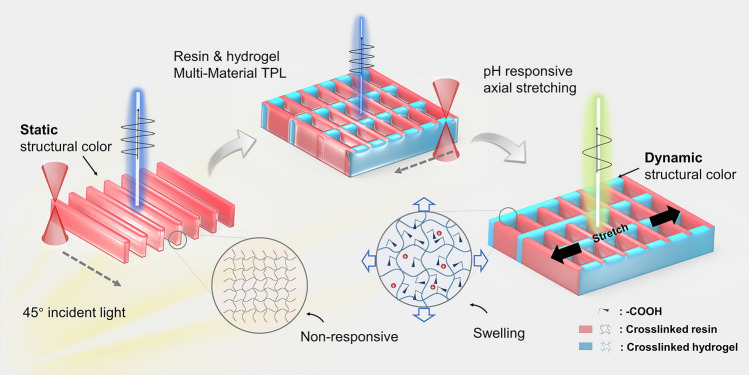

## Introduction

Structural coloration is a technique that manipulates light diffraction, scattering, and interference [1] to create color [2]. This is accomplished through the intricate interplay between polychromatic light and meticulously arranged micro- and nanoscale photonic structures. Structures capable of generating structural color are classified as photonic crystals (1D [3], 2D [4], and 3D [5, 6]) based on how the micro- and nanostructures are arranged periodically in space. Advanced mechanisms such as cavity resonance, surface plasma resonance, and Mie scattering have been intensively investigated to generate vivid colors [7]. For instance, metal structures that leverage plasmonic effects can produce colors with exceptional resolution and efficiency [8, 9]. In addition, the integration of Fabry–Pérot resonant cavities with grayscale lithography processes allows for the fine-tuning of color outputs by modulating the thicknesses of the dielectric layers [10, 11]. Furthermore, leveraging the chromatic polarization effect, 3D structural coloration can be achieved through a single-pulse ultrafast laser-induced micro-amorphous phase transition in lithium niobate [12, 13]. On the other hand, angle-independent structural color has been achieved through the controlled self-assembly of colloidal SiO_2_ nanoparticles onto highly aligned MXene films [14]. These cutting-edge approaches open new avenues for dynamic color displays [15–17] and information encryption [18–20], in addition to provide technical support for the development of tunable structural color devices.

Benefiting from stimulus-responsive deformable photonic nanostructures, dynamic structural colors can respond to external stimuli to display color variations [21]; such stimuli include temperature [22, 23], humidity [24, 25], pH [26], and electric field [27]. To date, dynamic structural color devices have attracted significant attention due to their high accessibility, direct detection and visualization capabilities, and their excellent sensitivity toward various external stimuli in practical applications. Representative examples include hydrogel dressings with photothermally responsive dynamic structural colors [28], tunable Fabry–Pérot (F–P) resonator consisting of a gas-responsive medium between a top disordered metal nanoparticle (MNP) layer and a bottom metal mirror for the detection of humidity and gases [29, 30], printable structural color inks exhibiting a thermal response [31], and tunable all-dielectric meta-surfaces for cryptographic applications and full-color reflective displays [32]. Furthermore, since the smallest structural units of structural colors are typically constrained to the visible wavelength scale, dynamic structural colors can be fabricated at the micrometer scale to address the limitations of conventional sensors that cannot be integrated into miniature devices due to size constraints [33]. This can enable non-contact sensing in microscale environments, such as in micro-robotics, microfluidics, and cellular biology. However, the application of dynamic structural color devices at the microscopic level is still hindered by the absence of high-precision bottom-up processing techniques and compatible structural designs.

Recent advancements in the miniaturization of tunable structural colors have been achieved using nanoscale additive manufacturing [34], particularly the two-photon polymer lithography (TPL) process [35–37]. For example, Yang et al. achieved reversible color display and hiding by heat-induced phase transitions of TPL-printed shape memory polymers [38]. Recently, hydrogel photoresists have been used as active materials for micro-dynamic structural color devices. More specifically, Marc et al. [39] used a cholesteric liquid crystals (LC)-based hydrogel resist to change colors within a limited range by altering the intrinsic periodicity of the chiral LCs. In another study, Chu et al. [40] used 4D-printed woodpile stimulus-responsive structural colors to prepare pixelated patterns, which could reversibly change color in response to variations in the solution pH. In a more recent report, Gu et al. [41] embedded periodic microspheres into a pH-responsive hydrogel through a combination of TPL and microsphere self-assembly to create dynamic structural colors with pH-sensing capabilities.

In contrast with the previous works reporting micro-dynamic structural colors (Table S1), our work investigates a transverse period-dependent color change mechanism that enables continuous and fast visual response upon deformation of the nanograting structure by swelling of the active material. More specifically, we present a 2.5-dimensional (2.5D) nanograting structure with heterogeneous materials and a method for creating and encoding dynamic structural colors. The impact of the nanogrid structure height and period on the structural color is systematically studied under normal and 45° incidence light. The grating structures are printed by interweaving a pH-responsive hydrogel with an IP-L photoresist. Transverse gratings printed with pH-responsive hydrogels can elongate the period of longitudinal grating in the swollen state, resulting in pH-tuned structural colors at a 45° incidence. Furthermore, a grayscale design approach based on 4D printing is developed to achieve the combined encoding of the structural color pattern by adjusting the height and period, wherein the bright stripe spacing in the grayscale image controls the grating printing period, and the grayscale value controls the grating height. This approach will be expected to significantly enhance the ability of spatial encoding and facilitate the precise printing of dynamic structural color patterns. Finally, the potential of micro-grating structure and this design strategy for use in structural color pattern printing, information encryption, and in situ sensing in microfluidic chips is evaluated.

## Experimental Section

### Preparation of the pH-Responsive Hydrogel Precursor

In a controlled environment illuminated with a yellow glow and maintained at a steady 25 °C, a precise assembly of chemicals was weighed out and combined in a 50 mL round-bottom flask: acrylic acid (AAC, 3 mL, 99%, Aikeshiji), N-isopropylacrylamide (NIPAAm, 4 g, 98%, Aladdin), ethyl lactate (EL, 2.5 mL, 98%, 9DingChemistry), and polyvinylpyrrolidone (PVP, 0.375 g, Mw 1,300,000, Aladdin). These components were meticulously weighed and combined, resulting in solution A after 12 h of continuous stirring. Subsequently, dipentaerythritol pentaacrylate (DPEPA, 0.4 mL, 98%, Aladdin), triethanolamine (TEA, 0.5 mL, 99%, Rhawn), 4-4′-bis(diethylamino)benzophenone (EMK, 100 μL, 97%, Aladdin)/ N,N-dimethylformamide (DMF, 99.5%, Rhawn) (20 wt%), and solution A (2.5 mL) were placed in another 50 mL round-bottom flask. The precursor of the hydrogel was obtained by stirring again at 1000 rpm for 12 h.

### Image Processing and Grayscale Design

All optical images were obtained through an imaging system consisting of objective len (Mitutoyo, Japan), CCD (HD206, AOSVI, China), light source, and optical components (LBTEX, China). The OpenCV (Open-source computer vision library) toolkit and C++ editor (Visual Studio 2022, Microsoft, USA) were used to process images and encod structural color patterns. Batch reading the optical images of structural color blocks and calculating the chromaticity distribution of colors in the HSV color space to obtain the chromaticity curve. Additionally, the average RGB values of structural color blocks with different parameters were calculated separately and combined with the corresponding height and period to form the color space.

The grayscale design method involves comparing the RGB values of the target pixel with the RGB values in the color space to find the closest structural color corresponding to the height and period. Then, using matrix operations, grayscale bar patterns are generated based on the height and period. The ratio of grayscale value to height is set at 10:1, and the ratio of pixels to period is 10:1. The created grayscale stripe pattern was imported into the Describe software (NanoScribe, Germany), mapping the grayscale values ranging from 0 to 255 to the height scale of 0–2.55 μm. The continuous mode of the Describe softwae can generate printing models with corresponding heights and periods based on the grayscale values and stripe periods of the grayscale stripe pattern.

### TPL of Grating Structure and Dynamic Grid Structure

Grating structures and dynamic grid structures were printed using TPL system (Photonic Professional GT2, NanoScribe GmbH, Germany) with a 63 × oil immersion objective (NA = 1.4, Zeiss, Germany). The grating structure was printed using the photoresist of IP-L (NanoScribe, Germany). The used optimal parameters include laser power of 25 mW, scan speed of 10 mm·s^−1^, hatching distance of 0.1 µm, and slicing distance of 0.1 µm. The printing is controlled by a code file generated by the software (Describe, NanoScribe, Germany) based on the grayscale. After printed, the glass sheet was immersed in isopropyl alcohol (IPA, 99.9%, Aladdin) for 30 min to remove the uncured photoresist. The glass sheet was then removed from the developer and dried naturally in air.

The TPL of dynamic grid structures utilizes a technology (multi-material stepwise polymerization two-photon lithography) we reported previously. At the center of the substrate, the hydrogel prepolymer was dropped again to completely submerge the grating structure. Using two reference points on the same horizontal line, rotate the current coordinate system to coincide with the coordinate system of the grating structure. Then, align the starting printing coordinates with the lower left corner of the grating array, thereby polymerizing the lateral grid of hydrogel material on the basis of the grating structure to form a dynamic grid structure. After printed, the glass sheet was immersed in isopropyl alcohol (IPA, 99.9%, Aladdin) for 30 min again to remove the uncured hydrogel.

### Preparation of pH Solution

Solutions with different pH values were obtained according to the acid–base dilution method, as follows. Firstly, calibrate the pH meter was calibrated using standard buffer solutions with pH values of 4.00, 6.86, and 9.18. NaOH powder (0.04 g, 96%) was added to deionized water (100 mL) under stirring to obtain an alkaline solution with a pH of ~ 12. Similarly, a 10% HCl solution (174 μL) was added to deionized water (50 mL) under stirring to obtain an acidic solution with a pH of ~ 2. These two solutions were then diluted separately to obtain range of acidic and alkaline solutions with pH values ranging from 2 to 12. A pH meter was used to determine the exact pH in each instance.

Two methods were employed to alter the pH value in the experimental demonstration. For the information encryption and microfluidic channel demonstration, solutions with pH values of 2 and 12 were rapidly added to deionized water through a dropper to change the pH value. For the color response demonstration of dynamic structural color devices, the dynamic structural color array was immersed in a pre-prepared solution with the desired pH value by replacing the solution. To minimize errors during the liquid exchange process, the replaced solution will be collected and remeasured after data collection.

### Preparation of the Microfluidic Channel

Templates for microfluidic channels were obtained by milling on PMMA plates using a desktop rotary engraver (DE-3, Roland, Japan) and a 0.6 mm milling cutter. PDMS (Sylgard 184, Dow Corning Corporation, USA) and vacuum oven (DZF-6012, Shanghai Yiheng Technical Co., Ltd., China) were mixed at 10:1 and stirred for 30 min and then placed in a vacuum oven and maintained under vacuum for 10–30 min to remove air bubbles. PDMS was poured onto the molds and heated at 80 °C for 2 h on a heating plate (PC-600D, Corning, USA). The cured PDMS was peeled off the molds to obtain PDMS films with microfluidic channel. The liquid was pumped into the microfluidic channel by pump (PHD 2000 Infusion, Harvard Apparatus, USA) at different flow rates.

## Results and Discussion

### TPL of Nanograting Structures and Generation of Static Structural Colors

**Fig. 1:**
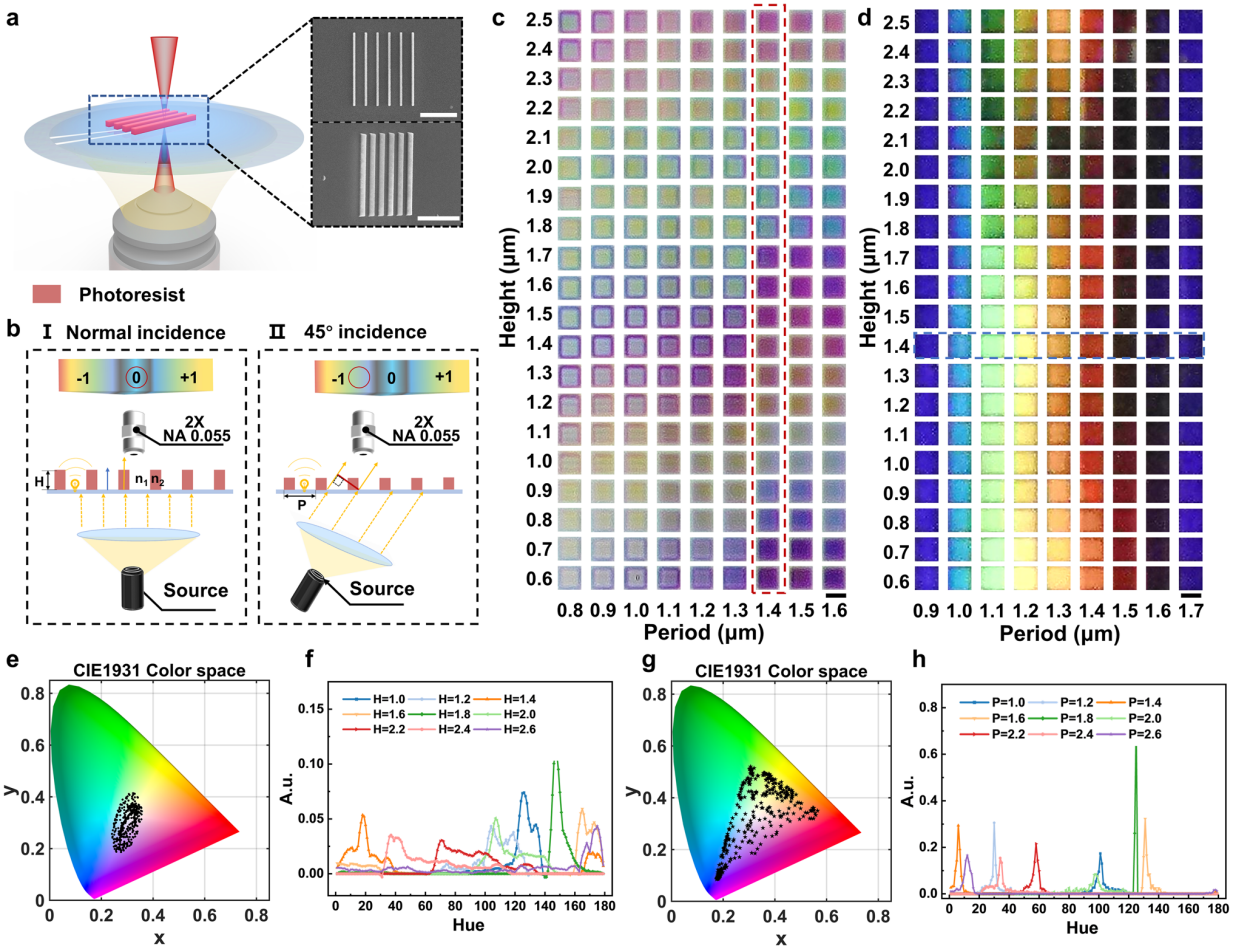
Structural color palettes of the nanogratings with different structural parameters.** a** Schematic diagram and SEM images of the TPL process used to prepare the nanograting with an IP-L photoresist. SEM images show a front view (top) and an oblique 45° view (bottom) of the printed nanograting structure. Scale bar: 10 μm. **b** Schematic representation of the structural color mechanism under (I) normal incidence and (II) oblique incidence light. **c** Optical images of the nanograting arrays with different heights and periods under normal light incidence. Scale bar: 20 μm. **d** Optical images of the nanograting arrays with different heights and periods when the light source was tilted at a 45° incidence. Scale bar: 20 μm. **e** The measured color coordinates of the structural colors shown in Fig. 1c are plotted as dots on the CIE 1931 color space chromaticity diagram. **f** Chromaticity histograms of the nanograting structures at different heights with a period of 1.4 μm. **g** Measured color coordinates of the structural colors shown in Fig. 1d are plotted as dots on the CIE 1931 color space chromaticity diagram. **h** Chromaticity histograms of the nanograting structures at different periods with a height of 1.4 μm

Raster diffraction is a key mechanism in the generation of structural colors. Using the IP-L resin as a photoresist, nanograting arrays with widths of 300–500 nm were processed using the TPL approach (Fig. 1a). To reduce the diffracted light reception, optical images resulting from the interactions between the raster arrays and the incident light at various angles were observed using an objective lens with a low numerical aperture (NA = 0.055) (Fig. 1b). When white light is vertically incident on the nanograting structure, the structure's height determines the phase change of light as it exits. Light passing through the grating interferes with light traveling through air, and constructive interference occurs when the phase difference between the two optical paths is an integer multiple of 2*π*. The wavelength of light that satisfies the interference condition can be estimated using Eq. 1 [42]:

1 $$\begin{array}{*{20}c} {m\lambda = (\eta_{1} - \eta_{2} )H} \\ \end{array}$$

where $\eta_{1}$ and $\eta_{2}$ represent the refractive indices of the photoresist ($\eta_{1}$ = 1.52), and the surrounding medium, *m*, is the diffraction order, $\lambda$ denotes the wavelength of light, and *H* is the height of the grating structure. Equation 1 shows that for the grating structure of a specific medium, the color observed under normal incidence is mainly determined by the height of the grating and the refractive index of the surrounding medium. Therefore, when the grating structure transitions from water ($\eta_{2}$ = 1.3) to air ($\eta_{2}$ = 1.0), its observed color also changes (Video S1). When white light is applied to the grating structure at an incident angle, objective lenses with small numerical apertures can observe the colors from non-zero-level diffraction spectra due to grating diffraction effects (Fig. 1bII). Variations in the period of the grating structure alter the optical distance between adjacent beams in the same observation direction, thereby affecting the shift in the diffraction spectrum, and ultimately altering the color of the observed structure. When white light is obliquely incident on the nanograting structure, the relationship between the angle of incidence, the angle of diffraction, the wavelength, and the period can be expressed in Eq. 2. In this case, only the wavelength of diffracted light collected by the microscope objective contributes to the color of the observed image. The condition is expressed in Eq. 3:

2 $$\begin{array}{c}\text{sin}\left({\theta }_{i}\right)-\text{sin}\left({\theta }_{d}\right)=\frac{m\lambda }{P}\end{array}$$

3 $$\begin{array}{c}\left|\text{sin}\left({\theta }_{d}\right)\right|\le NA\end{array}$$

where ${\theta }_{i}$ and ${\theta }_{d}$ represent the angle of incidence and the angle of diffraction, respectively, *m* is the diffraction order, λ denotes the wavelength of light, *P* denotes the period of the grating structure, and *NA* refers to the numerical aperture of the observation objective. Equations 2 and 3 indicate that when the incident angle and observation angle are fixed, the color observed under oblique 45° incidence is primarily determined by the grating period and the numerical aperture of the objective lens.

To study the interactions between the nanograting structures and the incident light, a grating array was printed with periods (P) ranging from 0.8 to 2.6 μm and heights (H) ranging from 0.6 to 2.5 μm, with a fixed laser power of 25 mW and scan speed of 10 mm·s^−1^ (Fig. S1). Figure 1c, d displays the color palettes observed for certain structural parameters under normal and oblique (45°) incidence light. Under normal incidence light, changes in H resulted in more significant color variations (Fig. 1c, red dashed box), whereas variations in P led to changes in the color saturation and chromaticity. Conversely, under oblique incidence light, changes in P caused greater color variations (Fig. 1d, blue dashed box), while variations in H had almost no impact on color. Since the transmission spectra were difficult to obtain owing to the customized observational conditions, the structural color was quantified using chromaticity value (hue) curves [43] based on the HSV color space (Fig. S2). Figure 1e shows the distribution of the entire color palette under normal incidence in the CIE diagram. For a period of 1.4 μm, the chromaticity coordinates of the structural colors originating from different heights are distributed along the periphery (Fig. S3a), and it can be seen that the chromaticity curve shifts periodically toward the red direction with an increasing height (Fig. 1f). Conversely, structural colors with the same height were clustered near the purple region (Fig. S3b), with peaks of the chromaticity curve being concentrated between 120 and 160 hue (Fig. S3c). Figure 1g depicts the distribution of structural colors in the color palette under 45° incidence, wherein the color coordinate distribution range covers the entire sRGB gamut. For a period of 1.4 μm, the chromaticity coordinates of structural colors with different heights gather in the red region (Fig. S3d), and the peak position of the chromaticity curve remains relatively stable (Fig. S3f). Meanwhile, for a height of 1.4 μm, the chromaticity coordinates of structural colors with different periods are distributed along the boundaries of the sRGB range, with the peak of the chromaticity curve periodically shifting toward the red direction (Fig. 1h). These results demonstrate that separately controlling the height and period of the grating structure allows for the color modulation of structural colors under normal and oblique 45° incidence.

### TPL of Multi-Material Nanogrids Exhibiting Dynamic Structural Colors

**Fig. 2:**
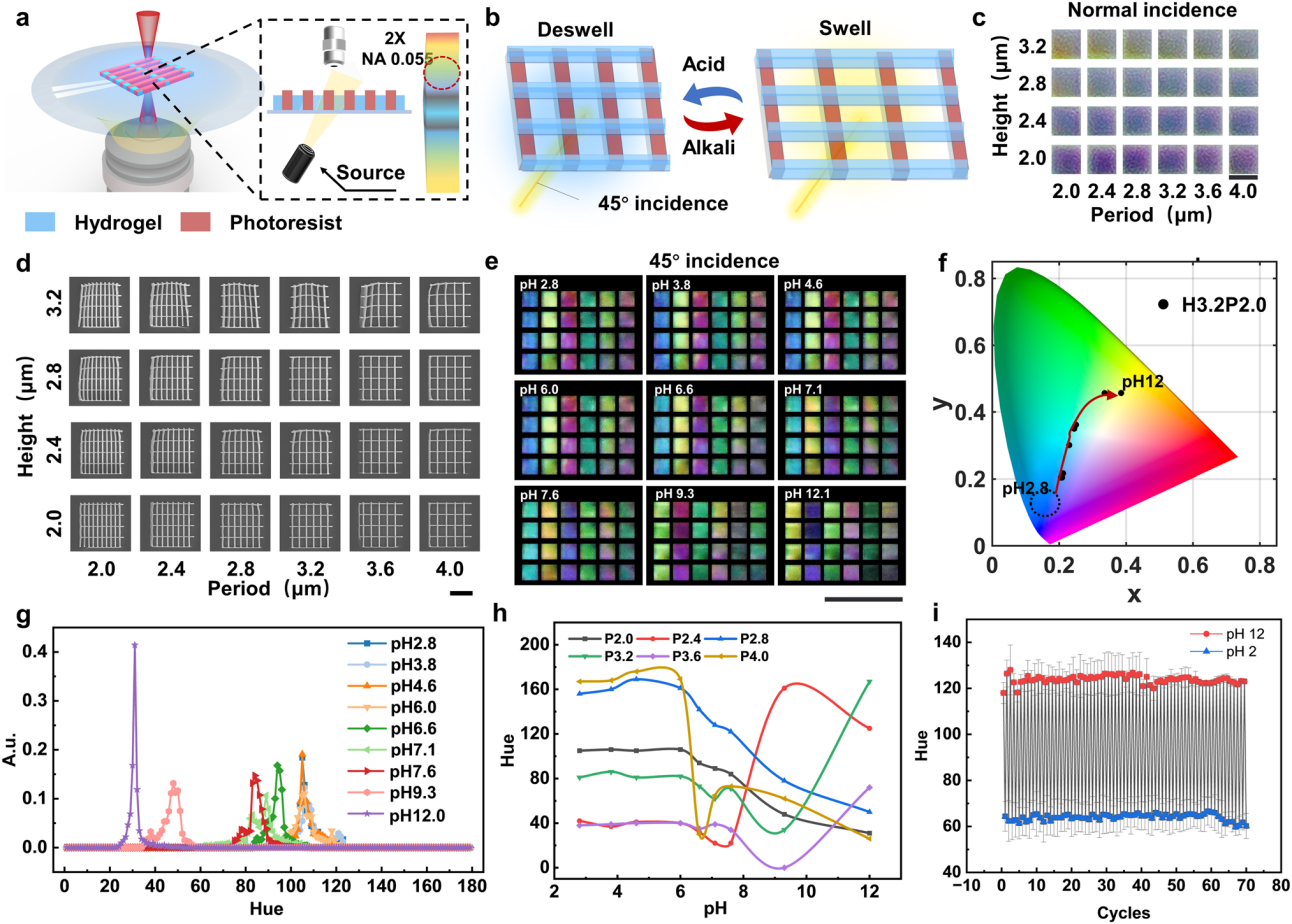
Preparation and pH-responsive performances of dynamic structural colors.** a** Schematic representation of the TPL process and observation conditions for the dynamic grid structures. Blue: pH-responsive hydrogel, red: IP-L photoresist. **b** Principle of color change in the dynamic grid structures. **c** Optical images of the dynamic grids with different heights (H) and periods (P) in deionized water under normal incidence light. Scale bar: 20 μm. **d** SEM images of the dynamic grids with different structural parameters. Scale bar: 10 μm. **e** Structural colors of the dynamic grids with different structural parameters and at different pH values under 45° incidence light. Scale bar: 100 μm. **f** Coordinates of the structural colors of a dynamic grid with *H* = 3.2 μm and *P* = 2 μm at different pH values on the CIE 1931 color space chromaticity diagram.** g** Chromaticity histograms of the structural colors of the dynamic grids with *H* = 3.2 μm and *P* = 2 μm at different pH values. **h** Peak values of the dynamic grid chromaticity histograms with different periods upon variation in the pH. **i** Changes of the hue peak of the dynamic grid with *P* = 2 μm when the environmental pH is switched between 2 and 12 over 70 cycles

In the above demonstration, a grating structure was used to generate static structural colors, and it was observed that the structural colors displayed at 45° incidence appeared more saturated. To create dynamic structural colors, a pH-responsive hydrogel was further polymerized in the direction perpendicular to the grating structure with a nominal period of 5 μm (Fig. 2a). This hydrogel was selected because the abundant carboxylic acid groups present in its acrylic acid-based internal network can swell and de-swell in response to changes in the surrounding pH, exhibiting a swelling ratio (rate of change of hydrogel length *λ* (*λ* = (*L* − *L*_0_)/*L*_0_)) of ~ 0.22 (Fig. S4). The vertical photoresist and horizontal hydrogel grating structures together formed a dynamic grid structure with pH responsiveness, wherein the vertical photoresist was used to generate structural colors, and the horizontal hydrogel grating was used to sense changes in the solution pH. As shown in Fig. 2b, when the pH in the solution reaches the response threshold of the hydrogel, the hydrogel begins to absorb water and swell. This action exerts a traction force on the longitudinal grating structure, causing an incremental lengthening of its transverse period. As presented in Fig. 1d and Eq. 2, under oblique incidence, the change in period will alter the color of the diffraction spectrum observed by the objective lens. Consequently, dynamic structural colors can be observed upon varying the pH (Video S2). This mechanism of adjusting the structural color by changing the grating structural period works only under oblique incidence (Video S3). Figure 2c shows the optical images of the grid structures with nominal heights ranging from 2.0 to 3.2 μm in deionized water under normal incidence conditions. Compared with the structural colors observed under and air environment, a lower sensitivity was observed with relation to the effects of height variations on the structural colors in deionized water. This was attributed to the fact that the refractive index difference between the IP-L photoresist and air (0.52) is significantly larger than that between the photoresist and water (0.22). Figure 2d shows the scanning electron microscopy (SEM) images of the grid structures with different nominal heights and periods, while Fig. S5a shows the SEM images of the same grid structures scanned after tilting 45°. Figure S5b shows plots of the measured heights and the measured line widths of the vertical gratings (photoresist) against the nominal heights. It is evident that the linewidth of the grid structure remained at ~ 530 nm, showing no significant change upon increasing the nominal height. In addition, it was found that the measured height of the grid structure linearly increased with the nominal height, which became less than the nominal height by ~ 300 nm. Furthermore, Fig. S5c presents plots of the measured grid structure periods and the measured linewidths of the horizontal gratings (hydrogel) against the nominal periods. As shown, the measured linewidth of the horizontal hydrogel grating increased gradually from 300 to ~ 400 nm as the nominal period increased. Moreover, the measured period of the grid structure was consistent with the nominal period.

To study the effect of the grid height and period on the dynamic structural color response, the grid array shown in Fig. 2d was sequentially immersed in HCl and NaOH solutions (pH 2.8–12.1), and optical images were recorded under 45° incidence light (Figs. 2e and S6). In all cases, the solutions were collected and recalibrated to obtain accurate pH values. To the naked eye, there was a noticeable color change in the grid structure as the pH was increased from 6 to 12. Moreover, the color changes were consistent in grid structures with the same period. Upon fixing the height and varying the period from 2.0 to 4.0 μm under different pH conditions, clear color changes were observed, as can be seen from the chromaticity coordinates in the CIE diagram (Fig. S7a–f). For grid structures with periods ranging from 2.0 to 2.8 μm, a wider range of colors was observed, in addition to a consistent shift in the CIE diagram as the pH was increased. Figure 2f illustrates the movement of the color coordinates of the grid structure (*P* = 2.0 μm) at different pH values. More specifically, between pH 2.8 and 6.0, blue colors are observed, while a further increase in pH led to the color gradually moving through the green region toward the yellow region. These observations were supported by measurement of the chromaticity values, wherein the peak of the chromaticity curve shifted from the blue region toward the red region with an increasing pH (Fig. 2g). This phenomenon is consistent with the previous observation that an increase in the period leads to a change in the structural color from blue to red. In addition, Fig. 2h shows the chromaticity peak values of the grid structures with periods of 2.0–4.0 under different pH conditions. It can be seen that between pH 2.8 and 6.0, the chromaticity peak values of all grid structures remained essentially unchanged. When the pH exceeded 6.0, the chromaticity peak values began to change; however, only the chromaticity curves of the grids where *P* = 2.0 and 2.8 μm exhibited monotonic decreases. Consequently, the pH sensitivity of the dynamic structural color for *P* = 2.0 and 2.8 μm was calculated to be ~ 12.5 and ~ 16.1 hue·pH^−1^, respectively. To characterize the response times of the dynamic structural colors and the interactions between the grid structure and the substrate, deformation of the grid structures under acidic to alkaline and alkaline to acidic conditions was recorded using a high-magnification (40×) inverted fluorescence microscope (Fig. S8a, c), respectively. Upon changing the solution pH from acidic to alkaline, the hydrogel began to swell and pull the longitudinal photoresist structure, which was detached from the substrate, leading to an increase in the period. Upon changing the solution pH from alkaline to acidic, the hydrogel began to de-swell, pulling the longitudinal photoresist structure and decreasing the period. The transverse hydrogel structure remained in contact with the substrate throughout this process and did not undergo flexural deformation. Figure S8b, d shows that the expansion time of the grid structure, ~ 5 s, is sufficiently larger than the contraction time, ~ 1 s. This may be due to weakening of the adhesion between the longitudinal photoresist structure and the substrate during the hydrogel swelling and deformation process.

Since the swelling of the hydrogel is reversible, the dynamic colors of grid structure are reusable. This potential was investigated further by repeatedly placing the grid structure array under pH 2 and 12 conditions for 70 cycles, and recording the peak of hue curves at each pH value (Fig. S9a). As shown in Fig. 2i, the peak value of the hue from the grid structure is relatively stable during the cycling test. The average value of hue is 123.9 under pH 12 condition with a standard deviation of 1.87 being obtained during the 70 cycles. In addition, upon increasing the number of cycles, the number of grid structures that lose their dynamic structural color gradually increased. After approximately 70 cycles, ~ 20% of the grid structures lost their structural colors (Fig. S9b).

### Patterned Direct Writing Based on the Grayscale Design Method

**Fig. 3:**
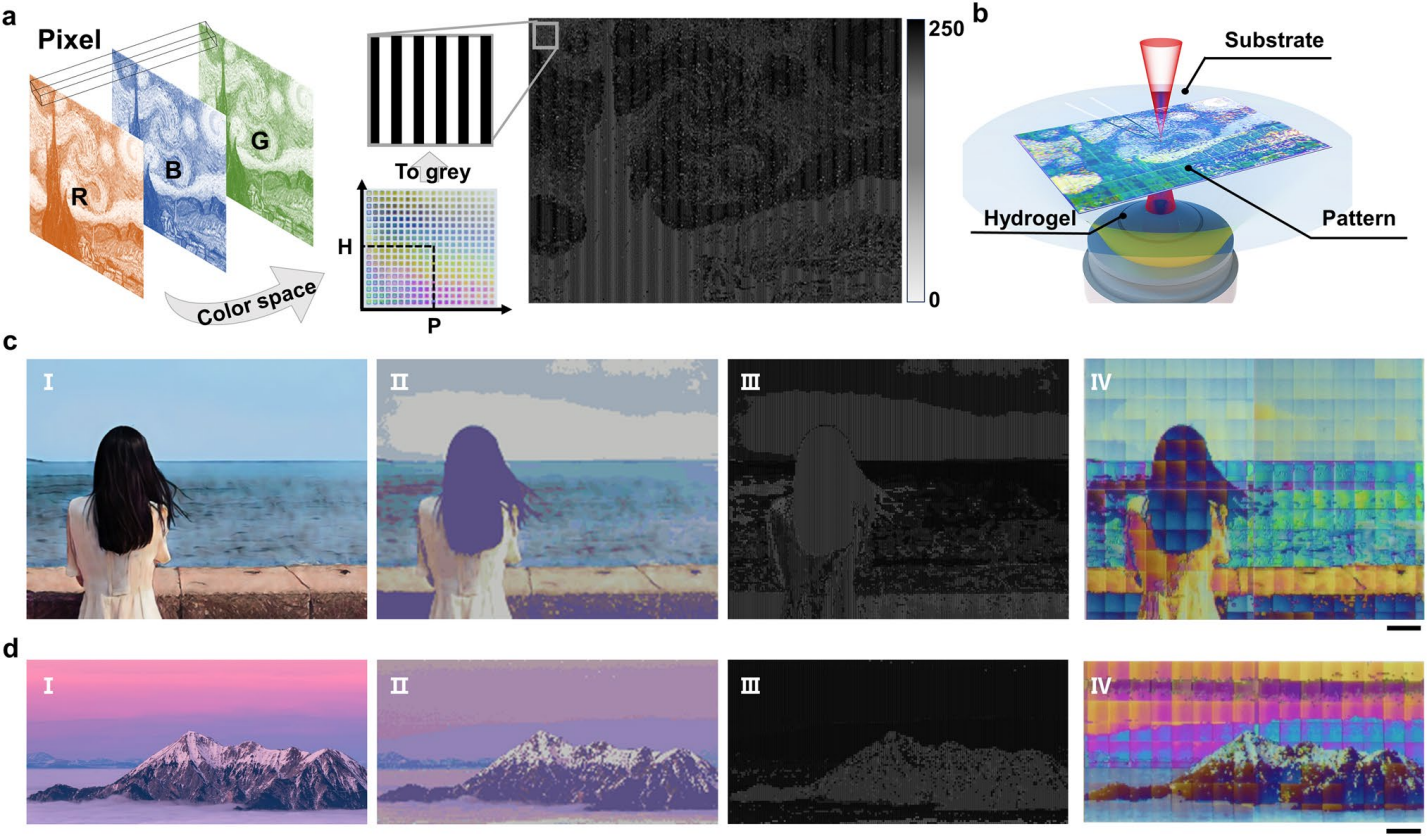
Grayscale grid structure design method and the patterned printing of structural colors.** a** Grayscale design process. A grayscale image generated based on RGB images was used to control the height and period of the grating structure in each pixel. **b** TPL of the structural color pattern based on the grayscale image. **c** Generation of a 3D-printed color photograph of a girl by the sea: (I) original photographic image, (II) simulated image generated using structural colors in the color space, (III) grayscale image generated using the grayscale design, and (IV) optical image of a structural color photograph printed from the grayscale image. Scale bar: 100 μm. **d** Generation of a 3D-printed color photograph of a mountaintop at sunset. Scale bar: 100 μm

To simplify the structural color design process and achieve color coding for any grid structure based on the color modulation effect of the grating period and height, a grayscale design method was proposed (Fig. 3a). This method converts a 2D color image into a grayscale striped image with alternating brightness and darkness characteristics to generate a 2.5D grating structure. Based on the RGB values of each pixel in the color image, the closest structural color was determined in the structural color space shown in Fig. S1b, and the corresponding heights and periods were obtained. Subsequently, a 5 μm wide square grayscale stripe was generated as a pixel, with the period set as the interval, and 10× the height set as the grayscale value. By traversing each pixel of the original image, a grayscale image containing information regarding the height and period of the grating structure was obtained. Consequently, the period and height of each unit in the image were encoded. Moreover, using the commercial slicing software Describe, the grayscale image was further transformed into a grating structure with a corresponding height and period, ultimately achieving patterned direct writing with encoded structures (Fig. 3b).

To demonstrate the encoding and control effects of the grayscale design method on the structural colors, a pattern was printed based on the famous oil painting "Starry Night" (Fig. 3b), along with two patterns printed based on real photographic images (Fig. 3c, d). The two printed images measured ~ 1 mm in length, reaching a point at which they were indistinguishable to the naked eye. Compared with the original photographic images, the simulated images exhibit a lower color saturation because they use the average values of the normal incident structural colors (Fig. 3cII, dII). The actual printed images are composed of writing blocks (60 μm × 60 μm), which are stitched together, as is evident by the noticeable stitching marks (Fig. 3cIV, dIV). In addition, certain color deviations are evident between the adjacent writing blocks, the printed pattern, and the original photo, which were attributed to the random deviation of the printed height of the raster structure from the nominal height. To measure the printing errors of the grating structures, grating arrays with a nominal height of 1 µm were printed. The 16 grating structures highlighted by red boxes in Fig. S10a were characterized using SEM to capture images at a 60° tilt angle. The actual heights were determined by measuring the heights in the image and dividing by sin(60°) (Fig. S10b). The average height error was ~ 0.1 µm, with an average chromaticity error of approximately − 3.1 hue. These printing errors may be caused by the laser power attenuation. Nonetheless, the effectiveness of the grayscale design method for encoding structural colors was experimentally verified, and the high-quality 3D printing of structural color nanopatterns was achieved.

### Information Encryption Based on Dynamic Structural Colors

**Fig. 4:**
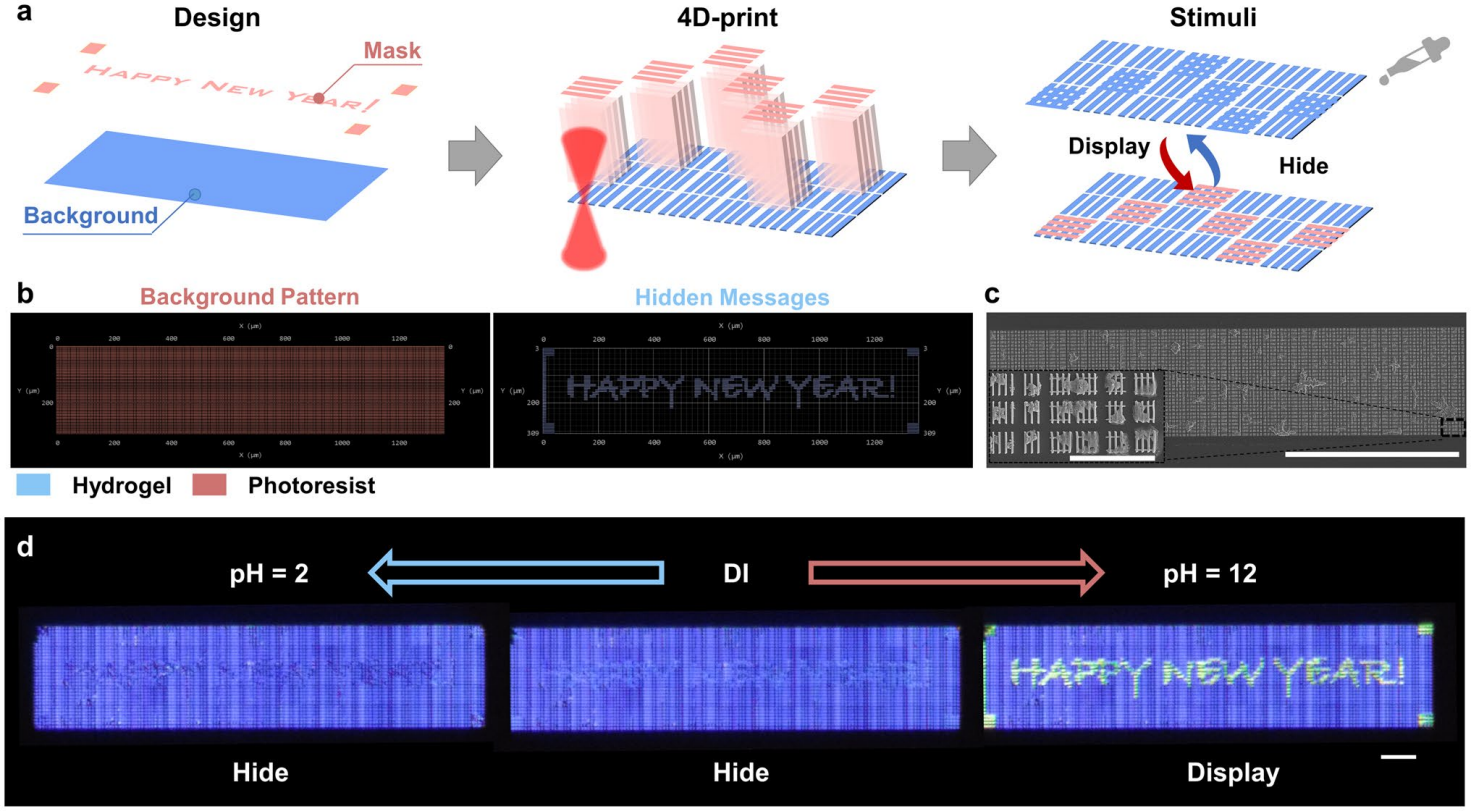
Programming strategy for displaying and hiding information.** a** Processing flow of the grid array with encrypted text information. The background color is composed of the vertical grid array of the photoresist material, while the hidden text information is composed of the horizontal grid of the hydrogel material and the vertical grid of the photoresist material. **b** Model of the grid array generated from the grayscale image. The left side shows the background pattern printed by the non-responsive photoresist, while the right side shows the hidden information printed by the pH-responsive hydrogel. **c** SEM image of the raster arrays containing hidden text information (scale bar: 500 μm) and a locally enlarged image (scale bar: 30 μm).** d** Optical images of the grid array with hidden text information, wherein the information is hidden in deionized water and acidic solution, but is displayed in an alkaline solution. Scale bar: 100 μm

The developed grayscale design method can also be used to program dynamic structural colors based on multi-material grid structures. By combining the characteristics of single-pixel encoding with the reversible properties of dynamic structural colors, both information display and concealment were achieved (Fig. 4a). Using the grayscale design method, a vertically striped grating structure with a corresponding height and period was generated based on a background pattern (Fig. S11a), and subsequently, a horizontally striped grating structure with a fixed period was created based on the desired pattern to be hidden (Fig. S11b). By leveraging the previously proposed multi-material TPL technology, a vertically striped grating structure was printed as the background using the non-responsive IP-L photoresist, and a horizontally striped grating structure with hidden information was printed on top using a responsive hydrogel to generate a dynamic grid structure at specific locations. When the pH of the solution was below the swelling threshold of the hydrogel, the pattern displayed only the background color under 45° incidence. However, when the pH exceeded the swelling threshold of the hydrogel, the horizontal grid expanded, stretching the period of the vertical grid, and creating a color change.

Figure 4b shows the array model generated from the grayscale image with the background pattern and the array model with the hidden message "HAPPY NEW YEAR!" Using TPL, the two patterns were overlaid and printed in the same rectangular array (Fig. 4c). The magnified SEM image shows that the background pattern is composed of vertical gratings, whereas the hidden message part consists of a combination of horizontal and vertical gratings that form grid structures. Figure S12 shows the results of hiding and revealing the information using two-color combinations. More specifically, in deionized water, the information was completely hidden, with the *P* = 2.0 μm array showing a green color, and the *P* = 2.3 μm array displaying an orange color. Upon the addition of an alkaline solution (pH 12), swelling of the hydrogel caused an increase in the period of the vertical grid, leading to a red shift in the structural color and gradually revealing the hidden text information. The subsequent addition of an acidic solution reversed this process, causing the hydrogel to de-swell, and pulling the vertical grids back to their original positions, hiding the information. However, owing to the smaller volume of the hydrogel in a strongly acidic solution (c.f., that in deionized water), the period of the vertical grid did not return to its original position but shrunk slightly. Therefore, under strongly acidic conditions, the information in the array still leaves a faint trace. Figure S8b shows the variation in the vertical grating length, wherein it can be seen that the length is ~ 0.9 μm smaller under acidic conditions, compared to in deionized water, i.e., the period is reduced by ~ 100 nm. To ensure that the structural period of the hidden pattern coincides with the structural period of the background pattern at pH 2, the structural period of the background pattern was set to 1.9 μm during the design process, and the period of the hidden structure was set to 2.0 μm. As shown in Fig. 4d and Video S4, the compensated structure can realize complete hiding of the information under acidic conditions.

### Integration of Dynamic Structural Colors in Microfluidics

**Fig. 5:**
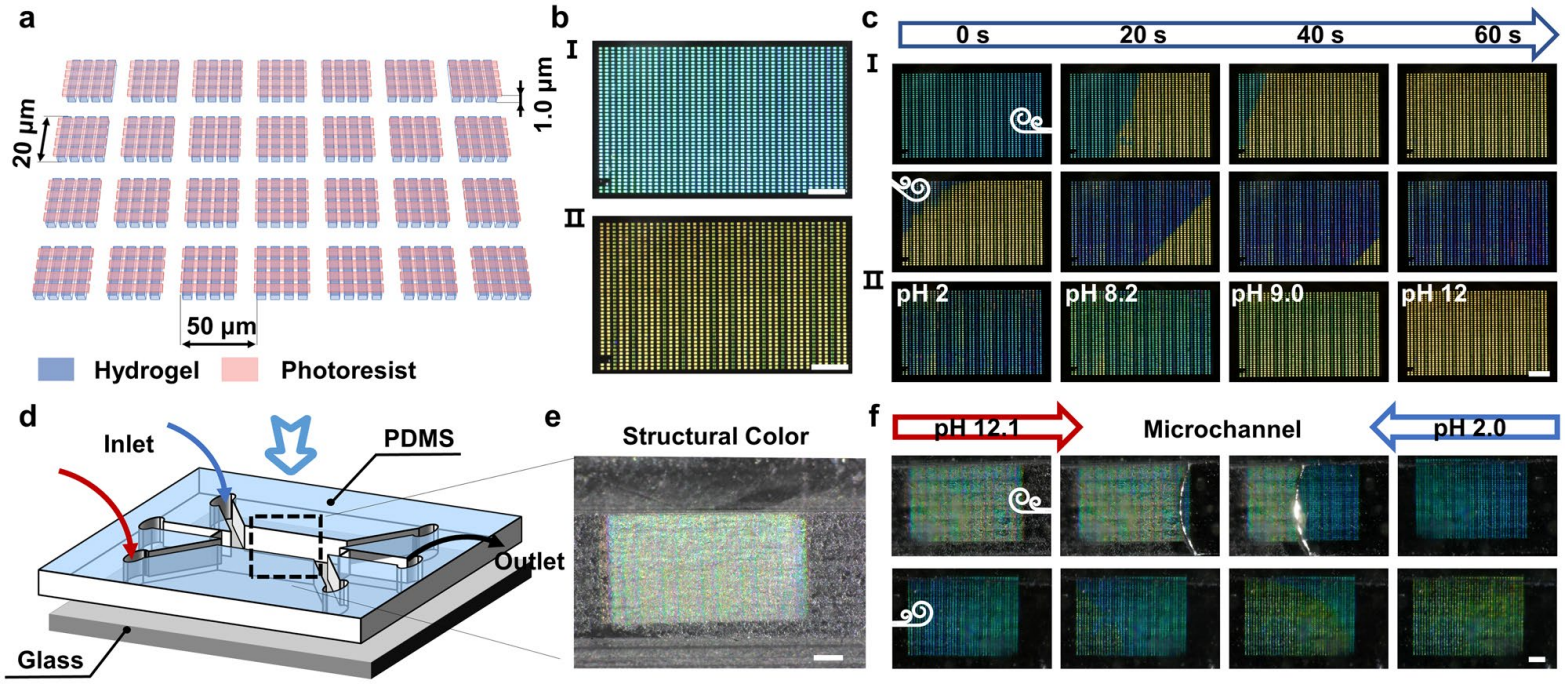
Dynamic structural color array for detecting the pH of the micro-environment.** a** Schematic diagram of a dynamic structured color array, with a single structured color unit length of 20 μm, a height of 2 μm, and an array period of 50 μm. **b** Optical images of the structured color array at 45° incidence: (I) structural color in deionized water and (II) structural color at pH 12. **c** Dynamic structured color array for the real-time monitoring of pH: (I) sensing the direction and velocity of the inflow of acidic and alkaline solutions and (II) color changes in solutions with pH values of 2, 8.2, 9.0, and 12. **d** Schematic representation of the integration of dynamically structured color arrays with microfluidic devices. **e** Partially magnified images of the microfluidic devices and optical images of the structural color arrays observed under air. Scale bar: 300 μm.** f** Image sequences of the dynamic structured color array integrated into the microfluidic device for pH monitoring. Scale bar: 300 μm

The grayscale design method can also be used to process large-scale dynamic grid arrays for in situ pH sensing (Fig. 5a). As shown in Fig. 5b, a sensor array composed of 40 × 40 independent grid units with a size of 2000 μm × 1200 μm was printed at the center of a glass substrate. Using the grayscale design method to control the period of the vertical grating of individual grid units at 2.0 μm, the grid array appears cyan colored in deionized water (Fig. 5bI) and orange–yellow in an alkaline solution (pH 12, Fig. 5bII). To demonstrate the visual sensing capability of the grid array for pH diffusion, a pH 12 NaOH solution and a pH 2 hydrochloric acid solution were slowly added dropwise to the right side and the upper left corner of the array, respectively (Fig. 5c and Video S5). As shown in the image sequence presented in Fig. 5cI, upon adding the alkaline solution (pH 12) to deionized water, the array gradually changed from cyan to orange–yellow (from right to left), while the addition of an acidic solution (pH 2) led to a gradual change from orange–yellow to deep blue (from the upper left to the lower right). Furthermore, by sequentially replacing the solution around the grid array with solutions with pH values of 2.5, 8.2, 9.0, and 12, the grid array exhibited intermediate colors between blue and orange–yellow (Fig. 5cII). Moreover, as shown in Fig. S13a, the structural colors of the grid structure arrays changed from blue to yellow when the arrays were placed in nine solutions with different pH from pH 2.6 to 10.8. Upon extracting the hue values of the grid structures at different pH values and fitting them according to the Boltzmann function (Fig. S13b), the obtained fitting curves allowed a qualitative estimation of the pH to be performed based on the hue values of the dynamic structural color arrays. These results indicate that the grid array can be used to visually sense the spatial diffusion direction, diffusion speed, and qualitative pH values of the microenvironment.

To demonstrate the functionality of in situ sensing by the microsensor, the grid array was further integrated into a microfluidic device (Fig. 5d). Using template replication of the engraved microfluidic channels to obtain a polydimethylsiloxane (PDMS) template, the diameter of the microchannel reached ~ 1500 μm (Fig. S14a, b). In this system, the PDMS template (Fig. S15a) was thermally bonded to a glass substrate printed with a grid array to form a complete microfluidic channel (Fig. S15b). Figure 5e shows an optical image of the solution intersection in the microfluidic channel and the integrated grid array within the microchannel. Using the microinjection system presented in Fig. S15c at a flow rate of 5 μL·min^−1^, solutions at pH 2 and 12 were pumped into the chip. As shown from Fig. 5f, a structural color change took place in the grid array, allowing the pH diffusion process to be observed in the microchannels. This 2.5D microscale sensing system not only provides a visual environmental sensing strategy, but it also holds the potential for integration within microfluidic devices due to the flexibility of the grayscale design approach developed herein.

## Conclusions

In this study, a 2.5D nanograting structure with heterogeneous materials and a grayscale design method suitable for combination with TPL were developed to construct dynamic structural colors. The grid structure was printed using a pH-responsive hydrogel and an IP-L photoresist via two-photon step-by-step multi-material lithography. The pH-responsive swelling characteristics of the hydrogel were used to alter the structural period of the longitudinal grating to change the dynamic structural color. To demonstrate its potential application, the dynamic structural color device was used for expressing and encrypting information, as well as for detecting pH changes in microfluidics. It was found that compared with the same type of micro-dynamic structure color mechanism, the dynamic structural color of the prepared heterogeneous material grating structure demonstrated an excellent comprehensive performance, including a high sensitivity (~ 12.5 hue·pH^−1^), a wide sensing range (pH 6–12), and an excellent patterned printing ability (Table S1). Although the structural colors generated based on grating diffraction effects are dependent on the viewing angle, it is easier to achieve specific irradiation and observation conditions at the microscale than at the macroscale by adjusting the microscopic observation system. Therefore, the prepared dynamic structural color device can maintain a good consistency in microenvironmental applications. Although only the pH-sensing function of the dynamic structural color array was demonstrated in this study, it is expected that humidity or temperature sensing capabilities could be achieved by replacing the stimulus-responsive polymers. Overall, this work provides a novel preparation approach and color-changing mechanism for miniature dynamic structural color sensors, which will greatly expand the application of structural color devices in microscopic fields, especially in the context of micro-robotics and microfluidics.

## Supplementary Information

Below is the link to the electronic supplementary material.

Supplementary file1 (DOCX 10249 kb)

Supplementary file2 (MP4 31692 kb)

Supplementary file3 (MP4 28194 kb)

Supplementary file4 (MP4 34025 kb)

Supplementary file5 (MP4 17738 kb)

Supplementary file6 (MP4 27053 kb)

Supplementary file7 (MP4 22190 kb)

## References

[1] X. Luo, D. Tsai, M. Gu, M. Hong, Subwavelength interference of light on structured surfaces. Adv. Opt. Photon. **10**, 757 (2018). 10.1364/aop.10.000757

[2] S. Kinoshita, S. Yoshioka, J. Miyazaki, Physics of structural colors. Rep. Prog. Phys. **71**, 076401 (2008). 10.1088/0034-4885/71/7/076401

[3] X. Zhu, J. Engelberg, S. Remennik, B. Zhou, J.N. Pedersen et al., Resonant laser printing of optical metasurfaces. Nano Lett. **22**, 2786–2792 (2022). 10.1021/acs.nanolett.1c04874 35311279 10.1021/acs.nanolett.1c04874

[4] X. Zhu, W. Yan, U. Levy, N.A. Mortensen, A. Kristensen, Resonant laser printing of structural colors on high-index dielectric metasurfaces. Sci. Adv. **3**, e1602487 (2017). 10.1126/sciadv.1602487 28508062 10.1126/sciadv.1602487 PMC5419704

[5] H. Liu, H. Wang, H. Wang, J. Deng, Q. Ruan et al., High-order photonic cavity modes enabled 3D structural colors. ACS Nano **16**, 8244–8252 (2022). 10.1021/acsnano.2c01999 35533374 10.1021/acsnano.2c01999

[6] H.K. Raut, H. Wang, Q. Ruan, H. Wang, J.G. Fernandez et al., Hierarchical colorful structures by three-dimensional printing of inverse opals. Nano Lett. **21**, 8602–8608 (2021). 10.1021/acs.nanolett.1c02483 34662137 10.1021/acs.nanolett.1c02483

[7] Z. Xuan, J. Li, Q. Liu, F. Yi, S. Wang et al., Artificial structural colors and applications. The Innovations **2**, 100081 (2021). 10.1016/j.xinn.2021.100081 10.1016/j.xinn.2021.100081 PMC8454771 34557736

[8] X.M. Goh, R.J.H. Ng, S. Wang, S.J. Tan, J.K.W. Yang, Comparative study of plasmonic colors from all-metal structures of posts and pits. ACS Photonics **3**, 1000–1009 (2016). 10.1021/acsphotonics.6b00099

[9] S. Zhang, Y. Wang, H. Wang, L. Zhong, X. Zhu et al., Laser writing of multilayer structural colors for full-color marking on steel. Adv. Photonics Res. **5**, 2300157 (2024). 10.1002/adpr.202300157

[10] J. Chen, G. Song, S. Cong, Z. Zhao, Resonant-cavity-enhanced electrochromic materials and devices. Adv. Mater. **35**, e2300179 (2023). 10.1002/adma.202300179 36929668 10.1002/adma.202300179

[11] Z. Yang, Y. Chen, Y. Zhou, Y. Wang, P. Dai et al., Microscopic interference full-color printing using grayscale-patterned fabry–perot resonance cavities. Adv. Opt. Mater. **5**, 1700029 (2017). 10.1002/adom.201700029

[12] J. Zhang, Z. Wang, B. Zhang, J. Qiu, Single-pulse-driven frame printing of chromatic pixels in lithium niobate crystal. Laser Photonics Rev. (2024). 10.1002/lpor.202400054

[13] Z. Wang, B. Zhang, Z. Wang, J. Zhang, P.G. Kazansky et al., 3D imprinting of voxel-level structural colors in lithium niobate crystal. Adv. Mater. **35**, e2303256 (2023). 10.1002/adma.202303256 37391205 10.1002/adma.202303256

[14] P. Xue, Y. Chen, Y. Xu, C. Valenzuela, X. Zhang et al., Bioinspired MXene-based soft actuators exhibiting angle-independent structural color. Nano-Micro Lett. **15**, 1 (2022). 10.1007/s40820-022-00977-4 10.1007/s40820-022-00977-4 PMC9705670 36441443

[15] R. Feng, H. Wang, Y. Cao, Y. Zhang, R.J.H. Ng et al., A modular design of continuously tunable full color plasmonic pixels with broken rotational symmetry. Adv. Funct. Mater. **32**, 2108437 (2022). 10.1002/adfm.202108437

[16] Y. Fu, C.A. Tippets, E.U. Donev, R. Lopez, Structural colors: from natural to artificial systems. Wiley Interdiscip. Rev. Nanomed. Nanobiotechnol. **8**, 758–775 (2016). 10.1002/wnan.1396 26952315 10.1002/wnan.1396

[17] L. Shang, W. Zhang, K. Xu, Y. Zhao, Bio-inspired intelligent structural color materials. Mater. Horiz. **6**, 945–958 (2019). 10.1039/c9mh00101h

[18] X. Hou, F. Vogelbacher, X. Lai, K. Li, Y. Song et al., Bioinspired multichannel colorful encryption through kirigami activating grating. Sci. Bull. **68**, 276–283 (2023). 10.1016/j.scib.2023.01.028 10.1016/j.scib.2023.01.028 36702683

[19] R. Li, K. Li, X. Deng, C. Jiang, A. Li et al., Dynamic high-capacity structural-color encryption *via* inkjet printing and image recognition. Adv. Funct. Mater. (2024). 10.1002/adfm.202404706

[20] W. Hong, Z. Yuan, X. Chen, Structural color materials for optical anticounterfeiting. Small **16**, 1907626 (2020). 10.1002/smll.201907626 10.1002/smll.201907626 32187853

[21] Y. Qi, S. Zhang, A.-H. Lu, Responsive structural colors derived from geometrical deformation of synthetic nanomaterials. Small Struct. **3**, 2270034 (2022). 10.1002/sstr.202270034

[22] J. Wang, Y. Sun, P. Jia, J. Su, X. Zhang et al., Wearable nanocomposite hydrogel temperature sensor based on thermally-switchable and mechanical-deformation-insensitive structural colors. Chem. Eng. J. **476**, 146602 (2023). 10.1016/j.cej.2023.146602

[23] I. De Bellis, D. Martella, C. Parmeggiani, D.S. Wiersma, S. Nocentini, Temperature tunable 4D polymeric photonic crystals. Adv. Funct. Mater. **33**, 2213162 (2023). 10.1002/adfm.202213162

[24] J.A.H.P. Sol, L.G. Smits, A.P.H.J. Schenning, M.G. Debije, Direct ink writing of 4D structural colors. Adv. Funct. Mater. **32**, 2201766 (2022). 10.1002/adfm.202201766

[25] J. Qian, S. Kolagatla, A. Pacalovas, X. Zhang, L. Florea et al., Responsive spiral photonic structures for visible vapor sensing, pattern transformation and encryption. Adv. Funct. Mater. **33**, 2211735 (2023). 10.1002/adfm.202211735

[26] J. Wang, Y. Hu, R. Deng, R. Liang, W. Li et al., Multiresponsive hydrogel photonic crystal microparticles with inverse-opal structure. Langmuir **29**, 8825–8834 (2013). 10.1021/la401540s 23768084 10.1021/la401540s

[27] L. Li, Z. Yu, C. Ye, Y. Song, Structural color boosted electrochromic devices: strategies and applications. Adv. Funct. Mater. **34**, 2311845 (2024). 10.1002/adfm.202311845

[28] L. Wang, X. Ding, L. Fan, A.M. Filppula, Q. Li et al., Self-healing dynamic hydrogel microparticles with structural color for wound management. Nano-Micro Lett. **16**, 232 (2024). 10.1007/s40820-024-01422-4 10.1007/s40820-024-01422-4 PMC11219637 38954118

[29] B. Ko, J. Kim, Y. Yang, T. Badloe, J. Park et al., Humidity-responsive RGB-pixels *via* swelling of 3D nanoimprinted polyvinyl alcohol. Adv. Sci. **10**, e2204469 (2023). 10.1002/advs.202204469 10.1002/advs.202204469 PMC9839877 36373672

[30] C. Jung, S.J. Kim, J. Jang, J.H. Ko, D. Kim et al., Disordered-nanoparticle-based etalon for ultrafast humidity-responsive colorimetric sensors and anti-counterfeiting displays. Sci. Adv. (2022). 10.1126/sciadv.abm8598 10.1126/sciadv.abm8598 PMC8916721 35275712

[31] Z. Zhang, C. Wang, Q. Wang, Y. Zhao, L. Shang, Cholesteric cellulose liquid crystal ink for three-dimensional structural coloration. Proc. Natl. Acad. Sci. U.S.A. **119**, e2204113119 (2022). 10.1073/pnas.2204113119 35639690 10.1073/pnas.2204113119 PMC9191658

[32] T. Badloe, J. Kim, I. Kim, W.-S. Kim, W.S. Kim et al., Liquid crystal-powered Mie resonators for electrically tunable photorealistic color gradients and dark blacks. Light. Sci. Appl. **11**, 118 (2022). 10.1038/s41377-022-00806-8 35487908 10.1038/s41377-022-00806-8 PMC9054757

[33] C.A. Koepele, M. Guix, C. Bi, G. Adam, D.J. Cappelleri, 3D-printed microrobots with integrated structural color for identification and tracking. Adv. Intell. Syst. **2**, 1900147 (2020). 10.1002/aisy.201900147

[34] Y. Li, M. Hong, Parallel laser micro/nano-processing for functional device fabrication. Laser Photonics Rev. **14**, 1900062 (2020). 10.1002/lpor.201900062

[35] W. Zhang, H. Wang, H. Wang, J. You En Chan, Q. Ruan et al., 2.5D, 3D and 4D printing in nanophotonics—a progress report. Mater. Today Proc. **70**, 304–309 (2022). 10.1016/j.matpr.2022.09.242

[36] Y. Liu, H. Wang, J. Ho, R.C. Ng, R.J.H. Ng et al., Structural color three-dimensional printing by shrinking photonic crystals. Nat. Commun. **10**, 4340 (2019). 10.1038/s41467-019-12360-w 31554803 10.1038/s41467-019-12360-w PMC6761189

[37] T. Mori, H. Wang, W. Zhang, C.C. Ser, D. Arora et al., Pick and place process for uniform shrinking of 3D printed micro- and nano-architected materials. Nat. Commun. **14**, 5876 (2023). 10.1038/s41467-023-41535-9 37735573 10.1038/s41467-023-41535-9 PMC10514194

[38] W. Zhang, H. Wang, H. Wang, J.Y.E. Chan, H. Liu et al., Structural multi-colour invisible inks with submicron 4D printing of shape memory polymers. Nat. Commun. **12**, 112 (2021). 10.1038/s41467-020-20300-2 33397969 10.1038/s41467-020-20300-2 PMC7782480

[39] M. Del Pozo, C. Delaney, C.W.M. Bastiaansen, D. Diamond, A.P.H.J. Schenning et al., Direct laser writing of four-dimensional structural color microactuators using a photonic photoresist. ACS Nano **14**, 9832–9839 (2020). 10.1021/acsnano.0c02481 32574044 10.1021/acsnano.0c02481 PMC7450659

[40] B. Liu, B. Dong, C. Xin, C. Chen, L. Zhang et al., 4D direct laser writing of submerged structural colors at the microscale. Small **19**, e2204630 (2023). 10.1002/smll.202204630 36382576 10.1002/smll.202204630

[41] K. Liu, H. Ding, Z. Chong, Y. Zeng, Y. Niu et al., Direct laser writing photonic crystal hydrogel sensors for *in situ* sensing in microfluidic device. Chem. Eng. J. **482**, 148679 (2024). 10.1016/j.cej.2024.148679

[42] J.Y.E. Chan, Q. Ruan, H. Wang, H. Wang, H. Liu et al., Full geometric control of hidden color information in diffraction gratings under angled white light illumination. Nano Lett. **22**, 8189–8195 (2022). 10.1021/acs.nanolett.2c02741 36227759 10.1021/acs.nanolett.2c02741

[43] W. Zhang, L. Qiu, K.J. Shea, J. Fan, Y. Liu et al., Quantitative analysis of structure color of photonic crystal sensors based on HSB color space. ACS Appl. Mater. Interfaces **14**, 35010–35019 (2022). 10.1021/acsami.2c08431 35856715 10.1021/acsami.2c08431

