# Supplementary material for: Nanograting-Based Dynamic Structural Colors Using Heterogeneous Materials
Source: Nanomicro Lett. 2024 Nov 11;17:59. doi: 10.1007/s40820-024-01554-7 (PMC11554963; doi:10.1007/s40820-024-01554-7)
Supplement: Supplementary file 1 — Supplementary file1 (DOCX 10249 kb) [file 40820_2024_1554_MOESM1_ESM.docx]

Supporting Information for

**Nanograting-Based Dynamic Structural Colors Using Heterogeneous Materials**

Jingang Wang^1, 2^, Haibo Yu^1^, Jianchen Zheng^1, 2^, Yuzhao Zhang^1,2^, Hongji Guo^1^, Ye Qiu^1, 2^, Xiaoduo Wang^1^, Yongliang Yang^1^ and Lianqing Liu^1,^*

^1^ State Key Laboratory of Robotics, Shenyang Institute of Automation, Chinese Academy of Sciences, Shenyang 110016, P. R. China

^2^ University of Chinese Academy of Sciences, Beijing 100049, P. R. China

*Corresponding author. E-mail: [lqliu@sia.cn](mailto:lqliu@sia.cn) (Lianqing Liu)

**Supplementary Figures and Tables**


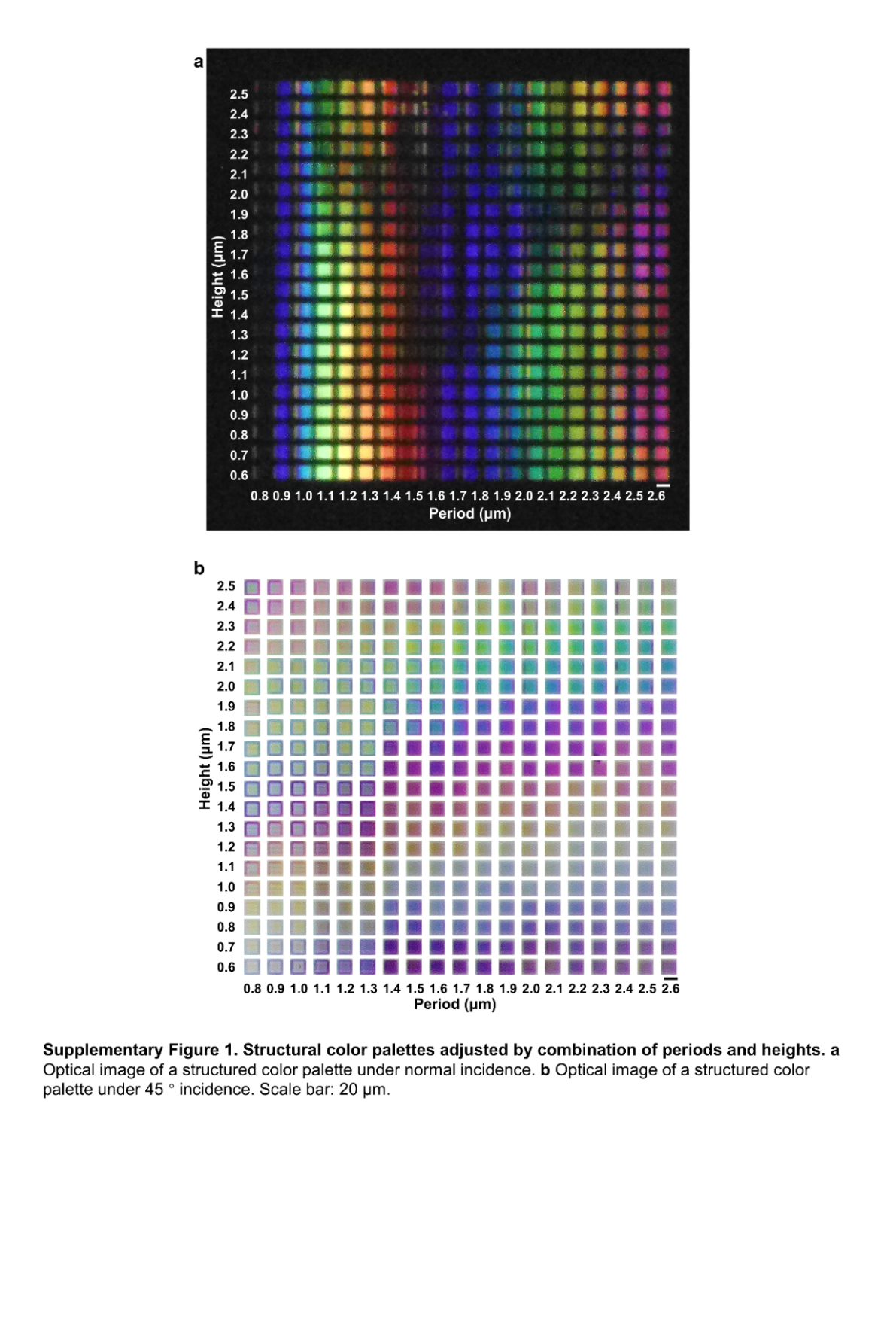


**Fig. S1 Structural color palettes adjusted by combination of periods and heights. a** Optical image of a structured color palette under normal incidence. **b** Optical image of a structured color palette under 45 ° incidence. Scale bar: 20 μm


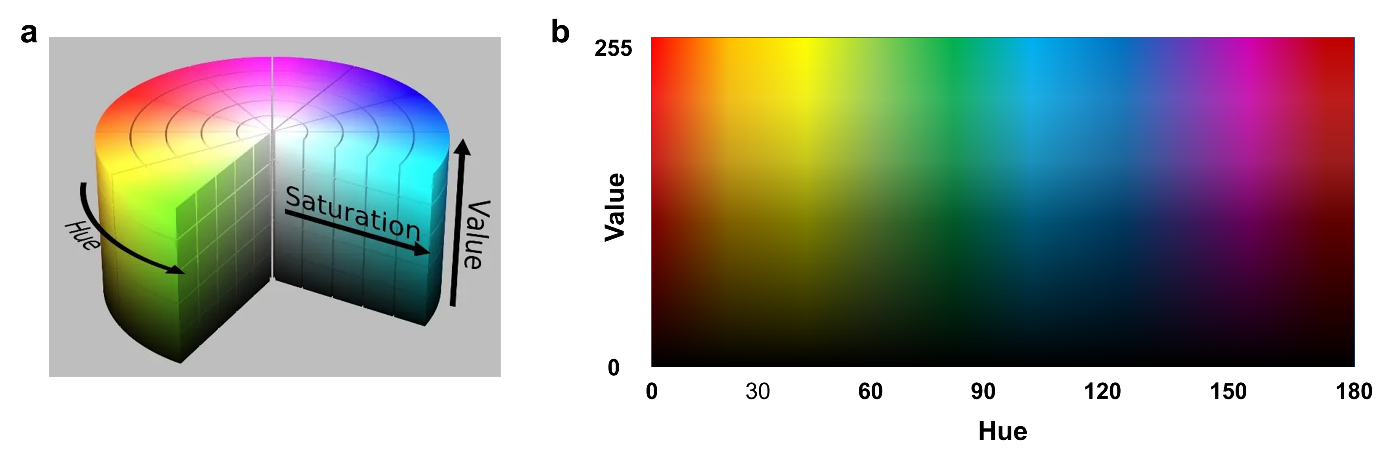


**Fig. S2 HSV color space. a** Schematic diagram of HSV color space. Each color is represented by Hue (H), Saturation (S), and Value (V). **b** In OpenCV, the unfolded graph of Hue and Value without considering Saturation. The H parameter represents color information, that is, the position of the spectral color, with a value range of 0 to 180. The range of V and S is 0-255


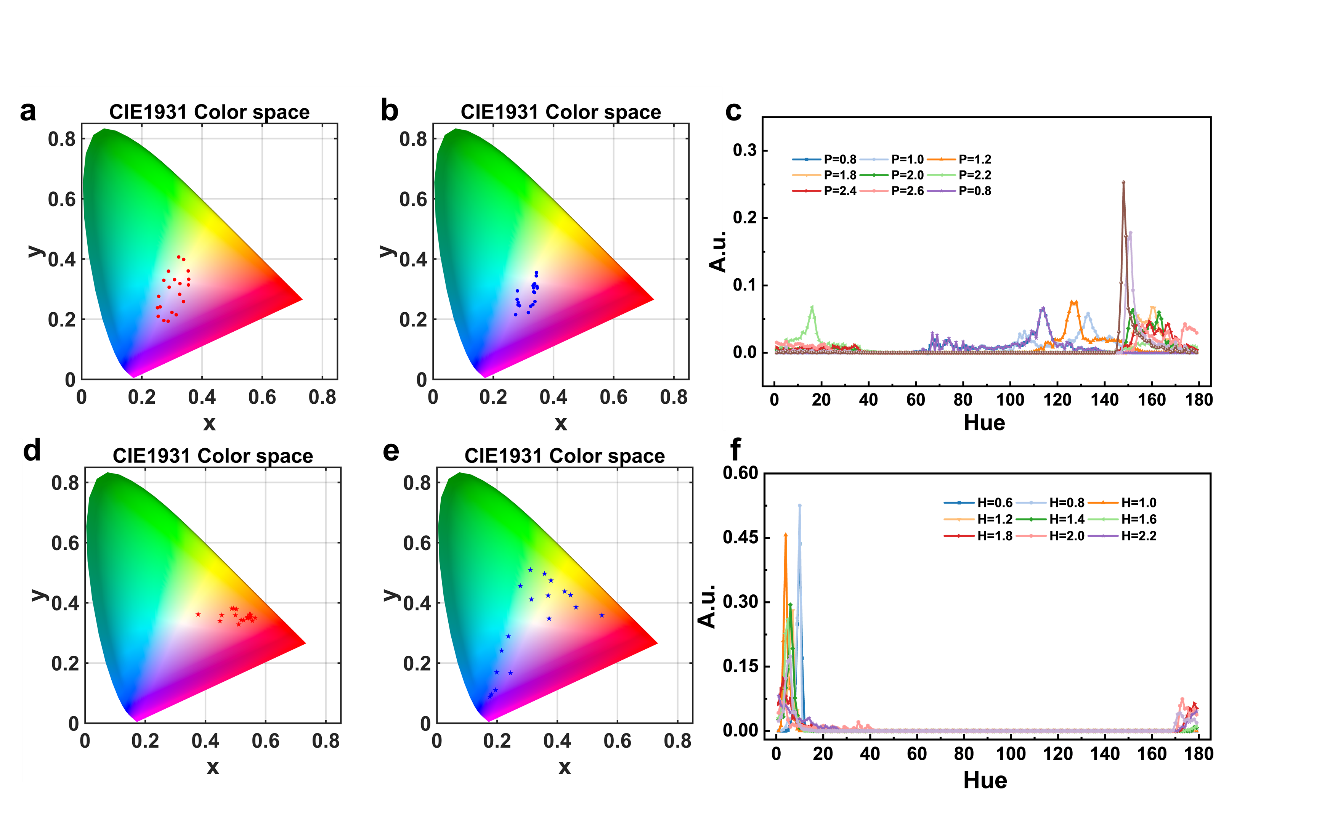


**Fig. S3 The influence of heights and periods on structural colors. a** Distribution of structural colors with different heights at a period of 1.4 μm under normal incidence in the CIE color space chromaticity diagram. **b** Distribution of structural colors with different periods at a height of 1.4 μm under normal incidence in the CIE color space chromaticity diagram. **c** Under normal incidence, the hue of structural colors with the height of 1.4 μm varies with the period. **d** The distribution of structural colors with different heights at a period of 1.4 μm under 45° incidence in the CIE color space chromaticity diagram. **e** Dstribution of structural colors with different periods at a height of 1.4 μm under 45° incidence in the CIE color space chromaticity diagram. **f** Under 45° incidence, the hue of structural colors with the period of 1.4 μm varies with heights


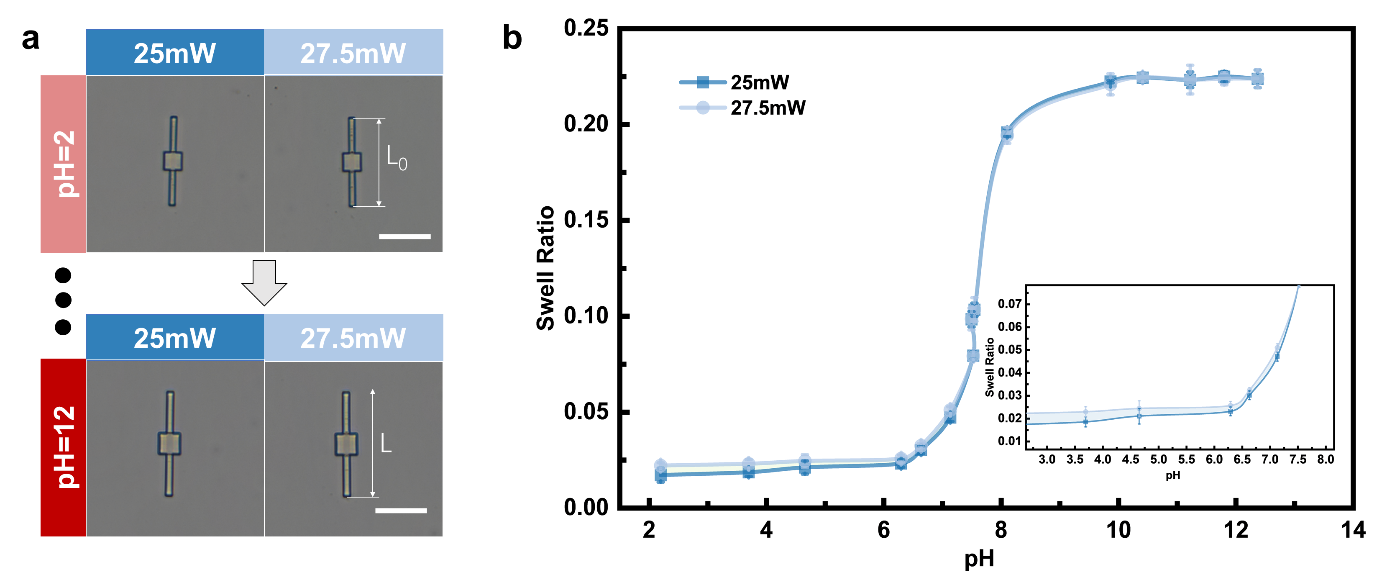


**Fig. S4 The swelling performance of hydrogel materials. a** Optical images of beam structures with nominal length (L_0_) of 50 μm printed using 25 and 27.5mW laser power respectively. Scale bars: 30 μm. **b** The swelling rate (the rate of change of body length λ (λ=(L-L_0_)/L_0_)) curve of hydrogel in different pH


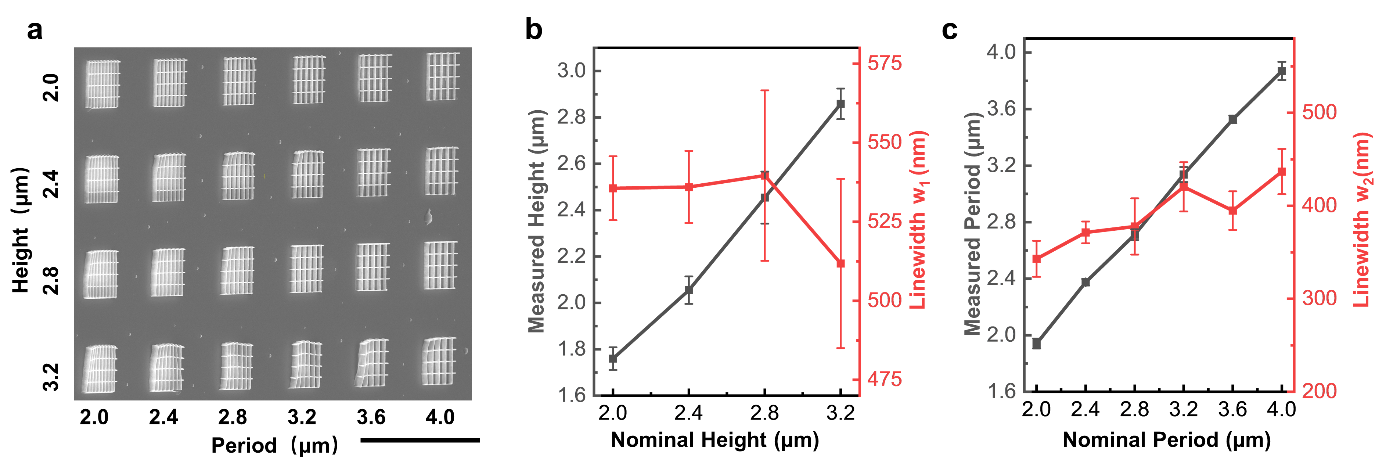


**Fig. S5 Scanning electron micrographs (SEM) of printed grid structures and measurement of height and period. a** SEM image of 45 ° tilted grid structures with heights from 2.0 μm to 3.2 μm and periods from 2.0 μm to 4.0 μm. Scale bar: 50 μm. **b** Measured grid linewidth w_1_ of vertical grating and measured height as a function of nominal height. **c** Measured grid linewidth w_2_ of horizontal grating and measured period as a function of nominal period


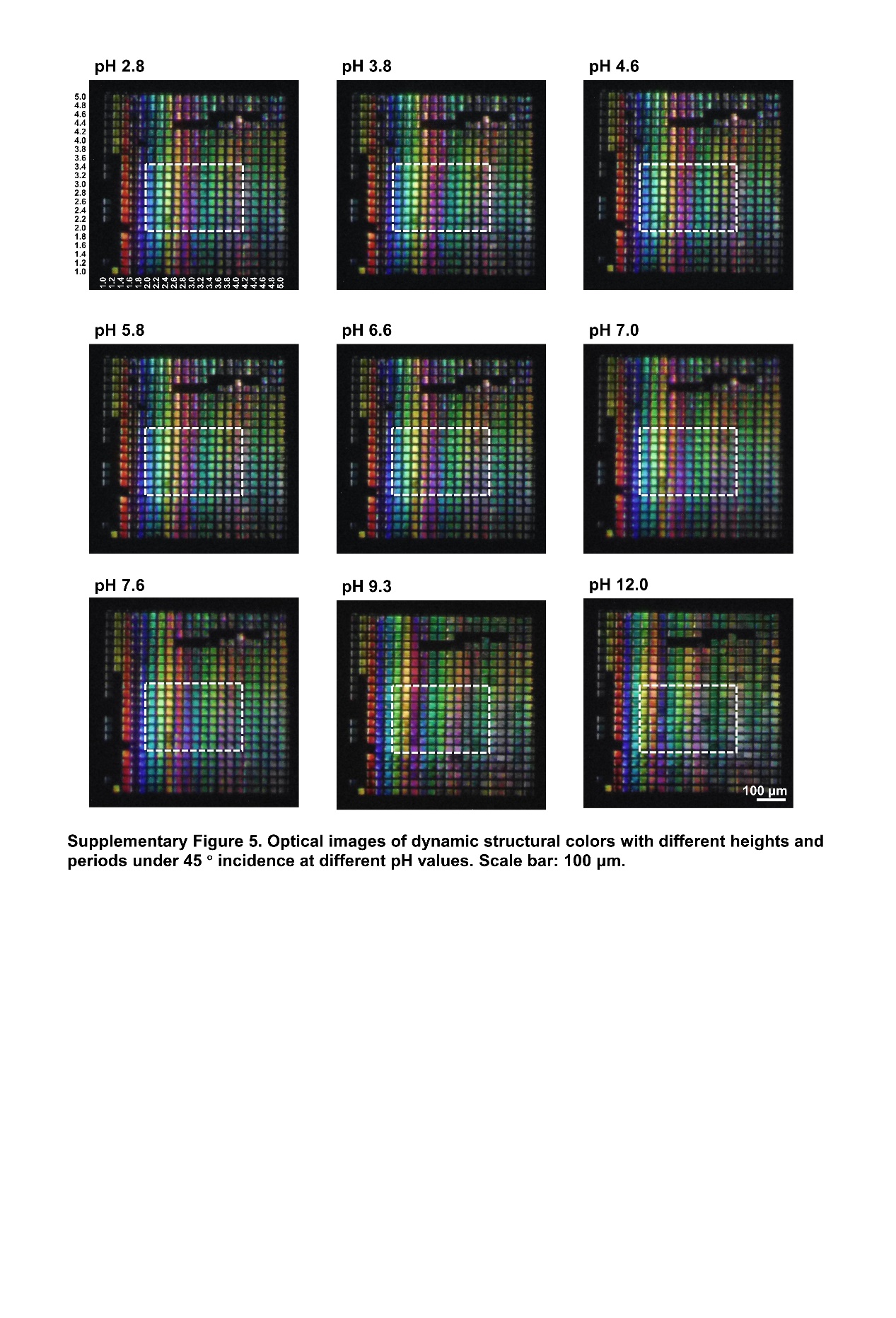


**Fig. S6** Optical images of dynamic structural colors with different heights and periods under 45 ° incidence at different pH values. Scale bar: 100 μm

**
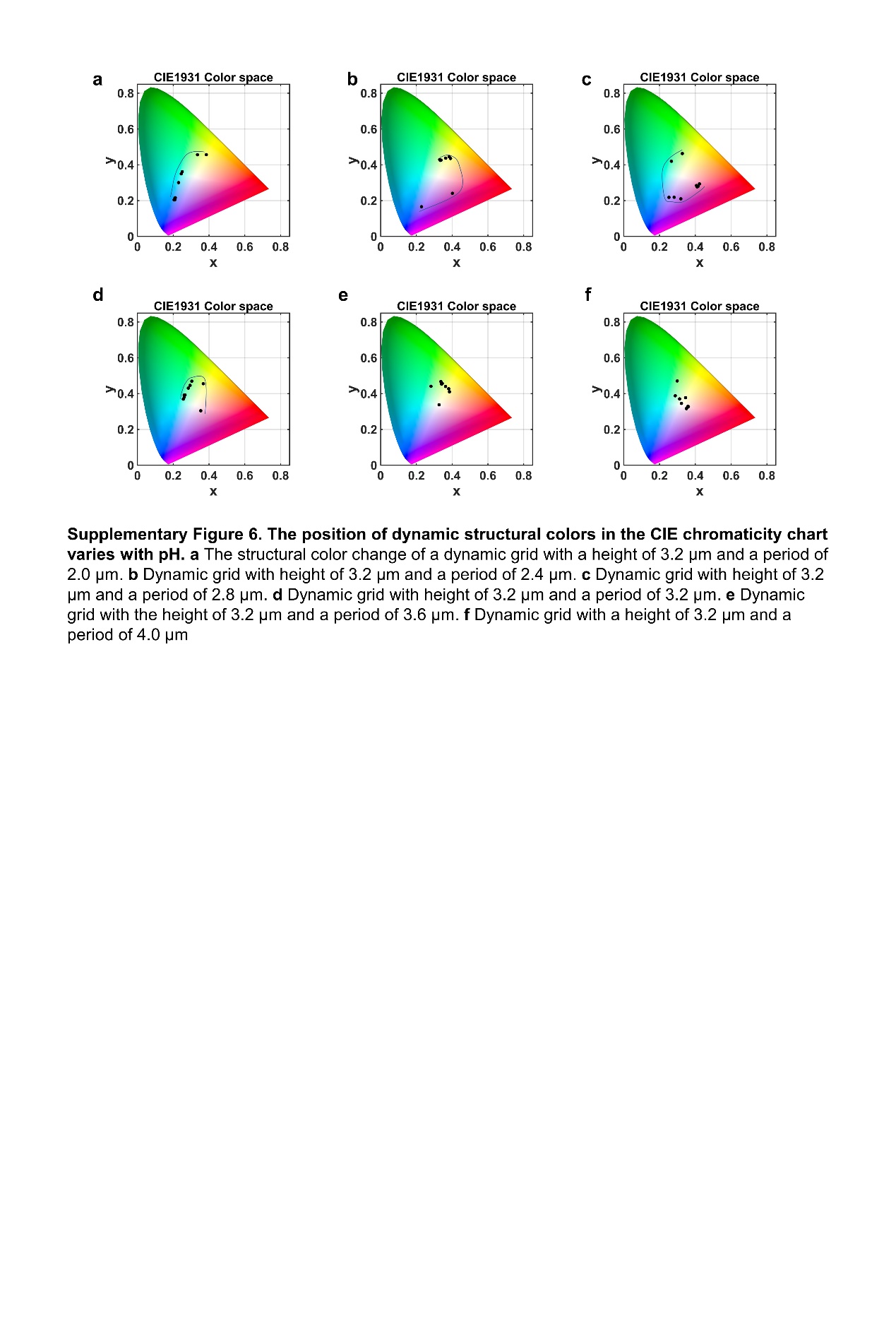
**

**Fig. S7 The position of dynamic structural colors in the CIE chromaticity chart varies with pH. a** The structural color change of a dynamic grid with a height of 3.2 μm and a period of 2.0 μm. **b** Dynamic grid with height of 3.2 μm and a period of 2.4 μm. **c** Dynamic grid with height of 3.2 μm and a period of 2.8 μm. **d** Dynamic grid with height of 3.2 μm and a period of 3.2 μm. **e** Dynamic grid with the height of 3.2 μm and a period of 3.6 μm. **f** Dynamic grid with a height of 3.2 μm and a period of 4.0 μm


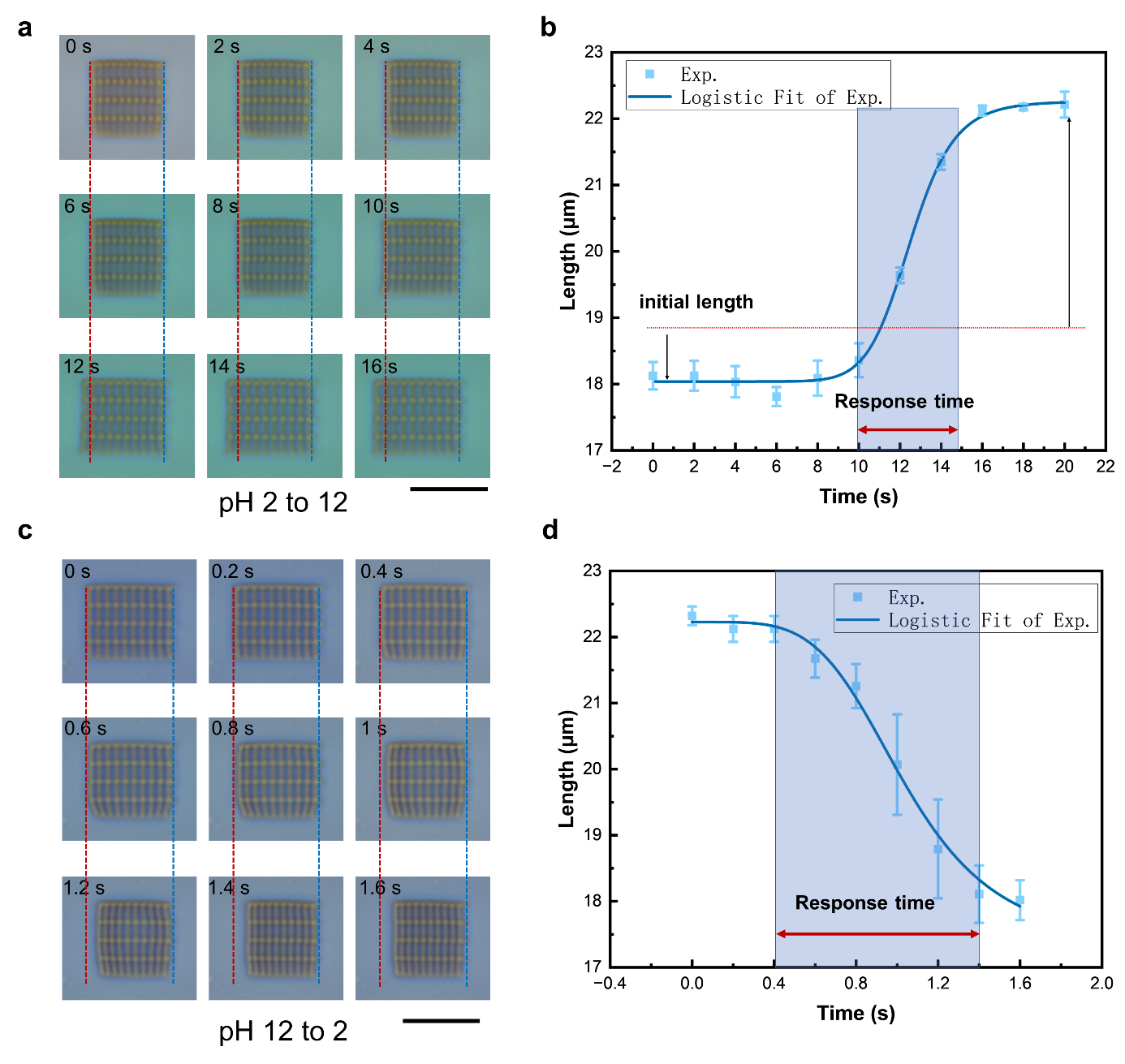


**Fig. S8 The deformation process of grid structure. a** Image sequences of deformation of the grid structures when the pH change from acid to alkaline. Scale bar:20 μm. **b** The variation curve of the horizontal body length of the grid structures over time from Fig. S8a. **c** Image sequences of deformation of the grid structures when the pH change from to alkaline to acid. Scale bar:20 μm. **d** The variation curve of the horizontal body length of the grid structures over time from Fig. S8c


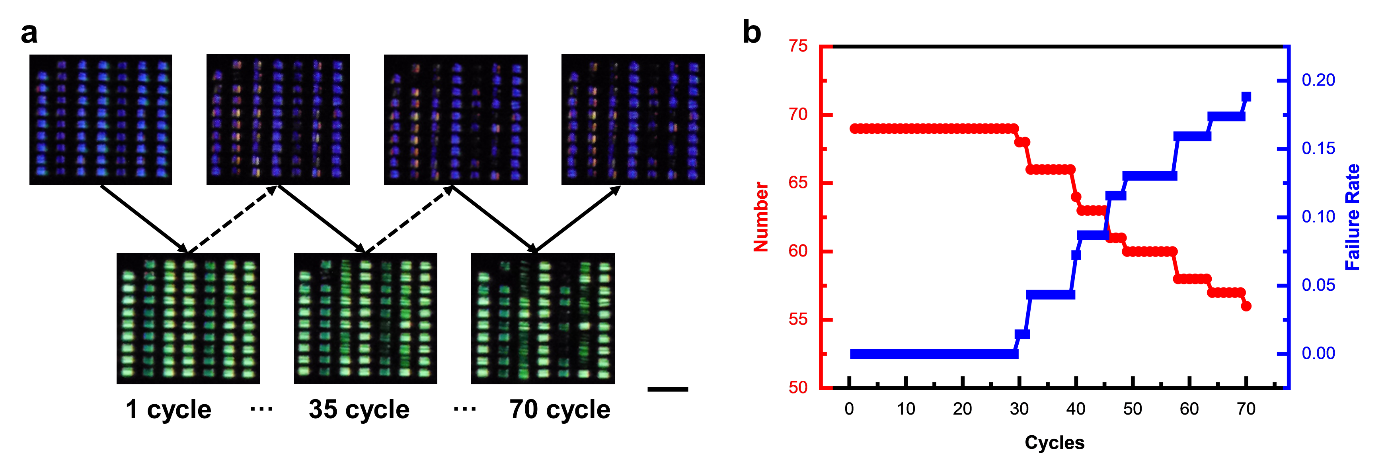


**Fig. S9 The stability of the dynamic structural colors over multiple working cycles.** **a** Optical images of grid structures array when environmental pH switches between 2 and 12 over 70 cycles. Scale bar: 100 μm. **b** Quantity change curves (red curves) and failure rate change curves (blue curves) for normal dynamic structural colors as the number of cycles increases


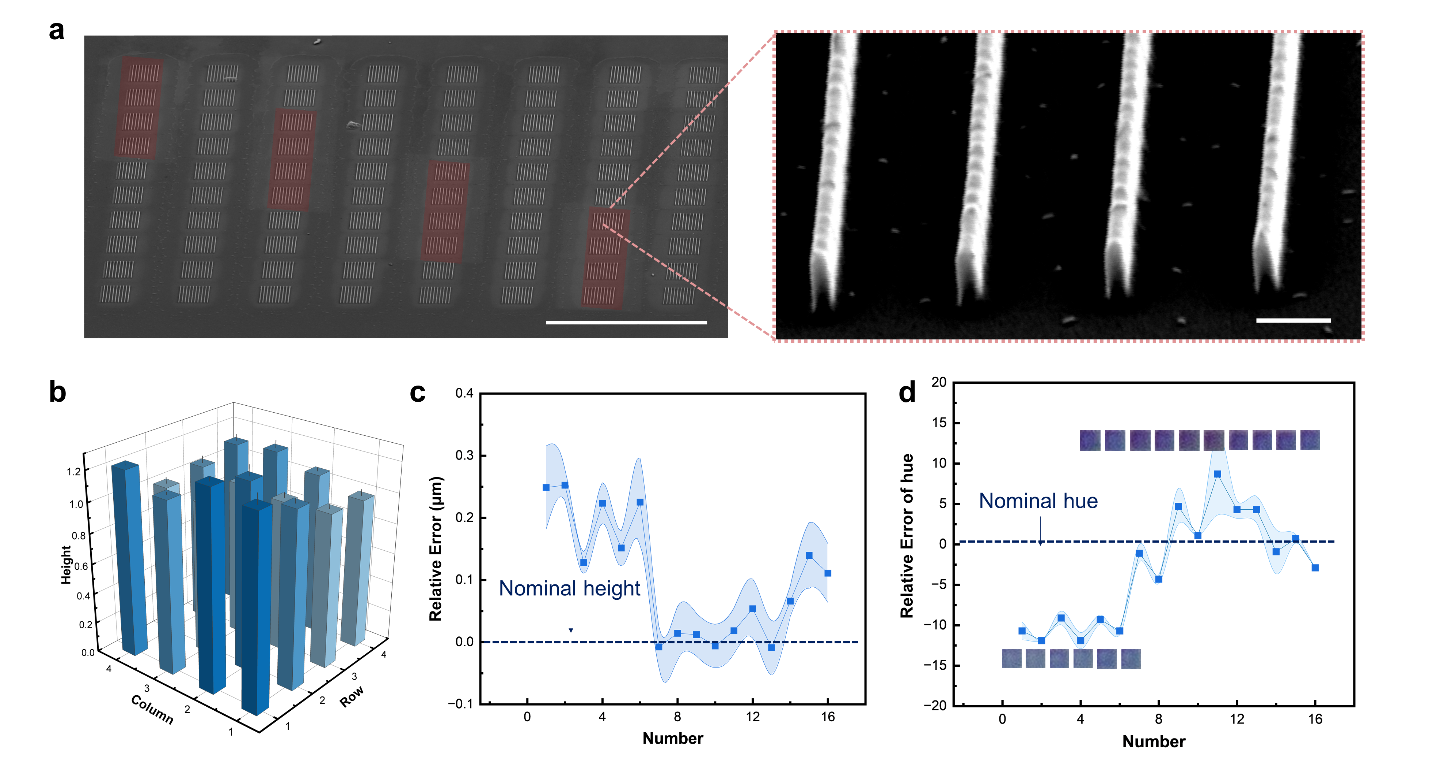


**Fig. S10 Printing error and chromaticity error of grating structures.** **a** SEM images of grating structures array with a nominal height of 1 µm, scale bar: 100 μm. and partial enlarged image of the end of the grating structure at a 60° tilt angle, scale bar: 1 μm. **b** Actual heights of the 16 grating structures highlighted in red boxes in Fig. S9a. **c** The error curve of the actual height relative to the nominal height. **d** The error curve of the actual hue relative to the nominal hue.


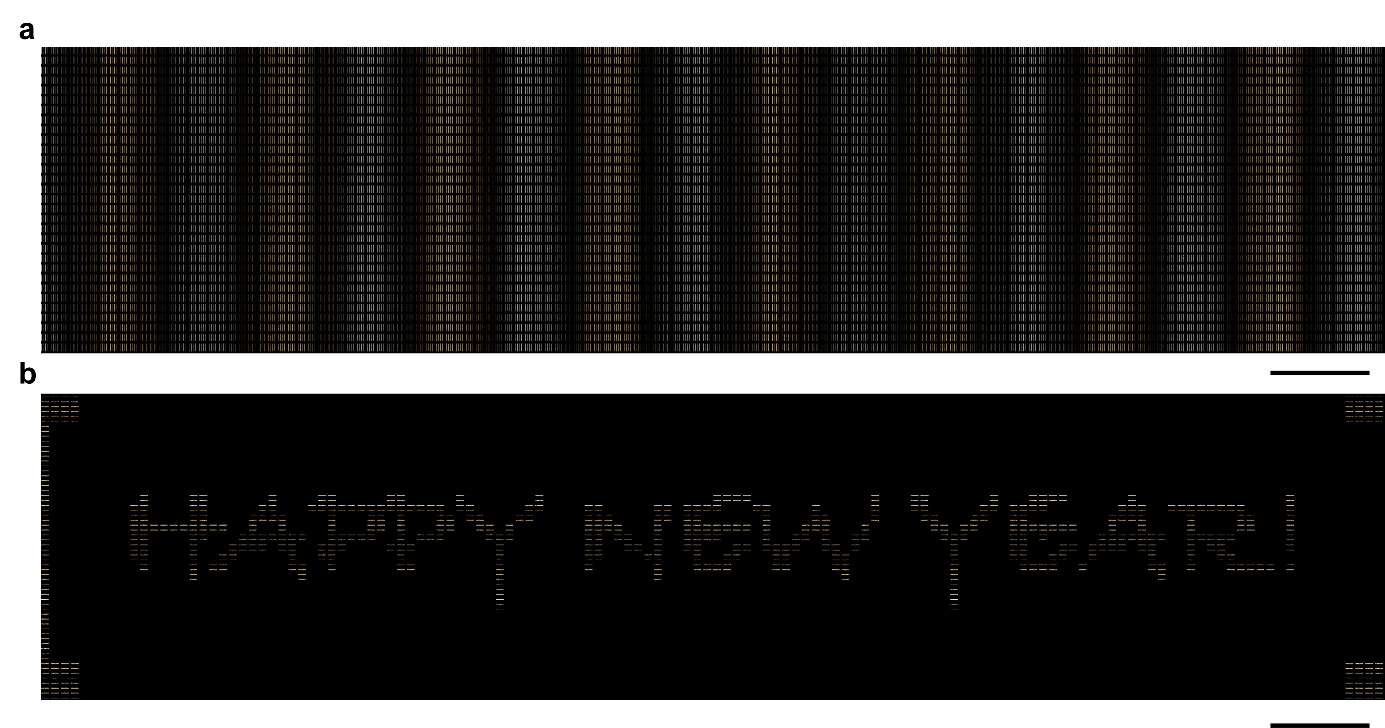


**Fig. S11 The grayscale design for displaying and hiding textual information. a** The grayscale image of the word "HAPPY NEW YEAR!" is composed of a horizontal grid, with a period of 5 μm and a height of 1 μm. **b** A grayscale image with a background color composed of vertical grids, with a period of 2 μm and a height of 1 μm. Scale bars: 100 μm


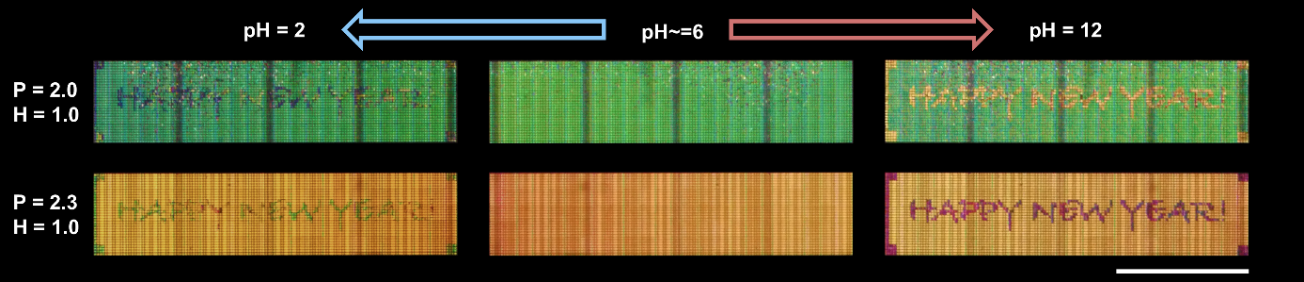


**Fig. S12** Optical images of unoptimized dynamic grid arrays hiding information in deionized water and displaying information in alkaline solution and leaving slight traces in acid solution


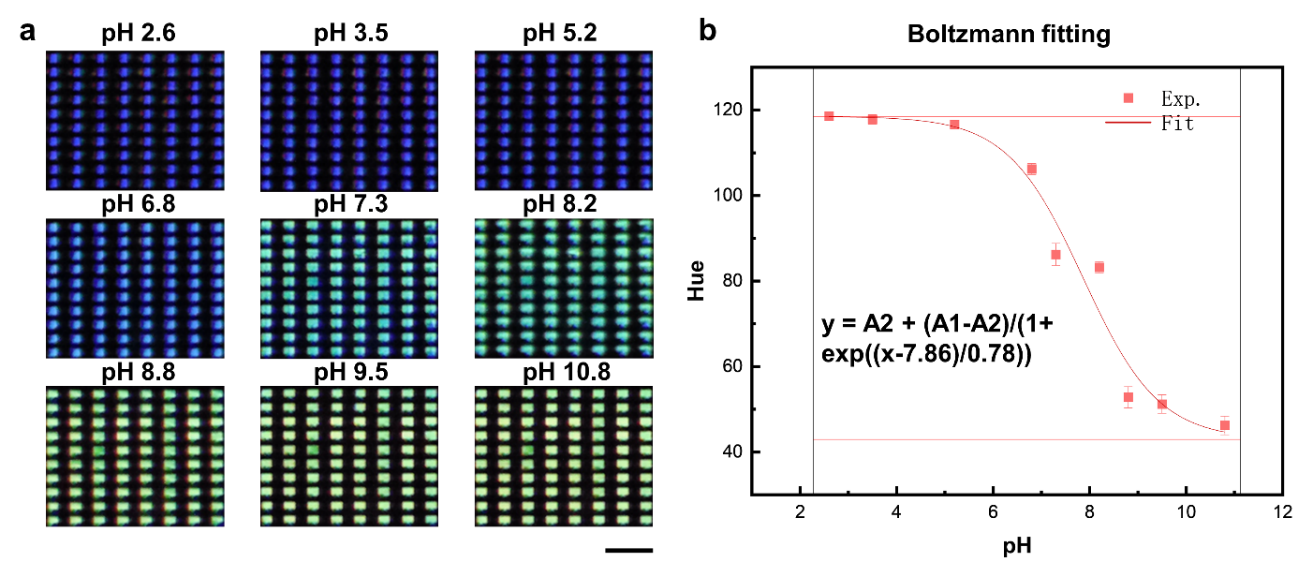


**Fig. S13 Structural colors of the grid structure arrays when the array was placed in nine solutions with different pH from pH 2.6-10.8. a** Optical images of structural color of the grid structure arrays when the array was placed in nine solutions with different pH from pH 2.6-10.8. Scale bar: 100 μm. **b** Average of hue values of the five grid structures in the array at different pH values and the fitting curves by Boltzmann function. A1 and A2 represent the maximum and minimum hue values of the structural colors within the interval pH 2-12, respectively.


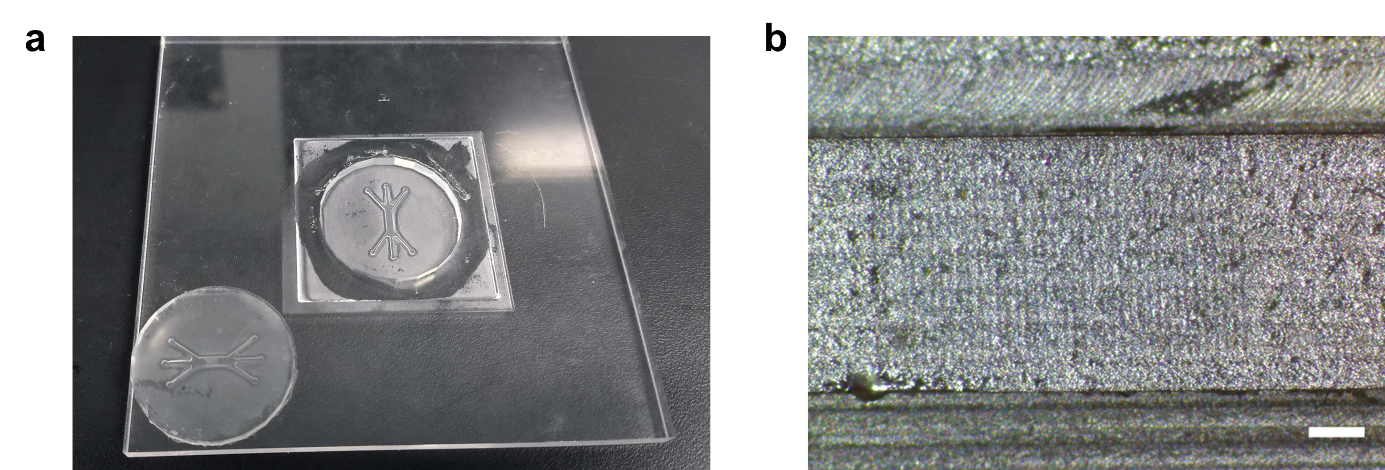


**Fig. S14 The preparation of microfluidic chips.** **a** Template replication of microfluidic chips. **b** Optical image of microfluidic channels. Scale bars: 300 μm


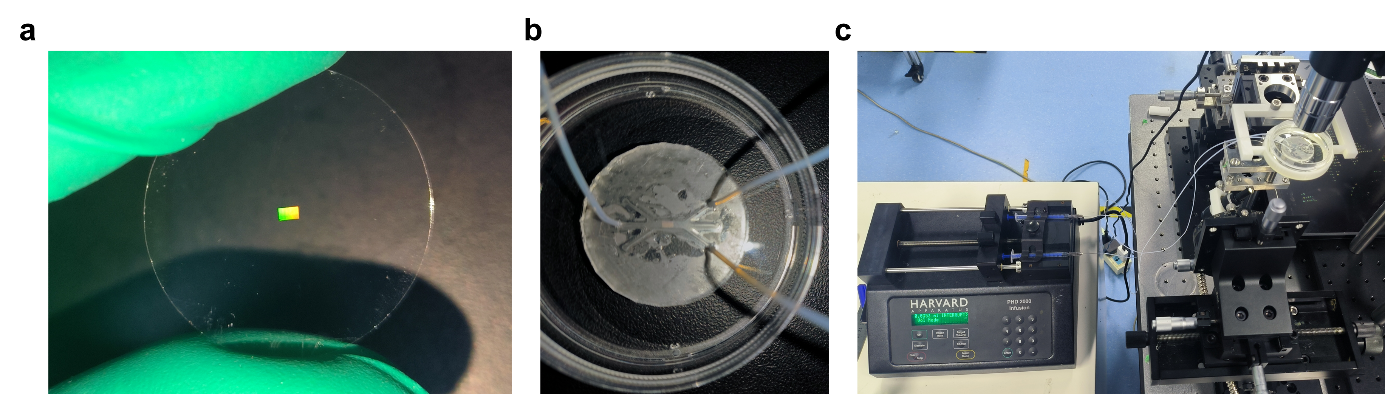


**Fig. S15 The integration of dynamic structured color arrays and microfluidic device. a** A photo of a printed dynamic structured color array. **b** A photo of the integration of a dynamic structured color array and microfluidic channels. **c** Photo of the micro injection pump and observation system

**Table S1** Summary of existing works of micro structures with dynamic structural colors

**
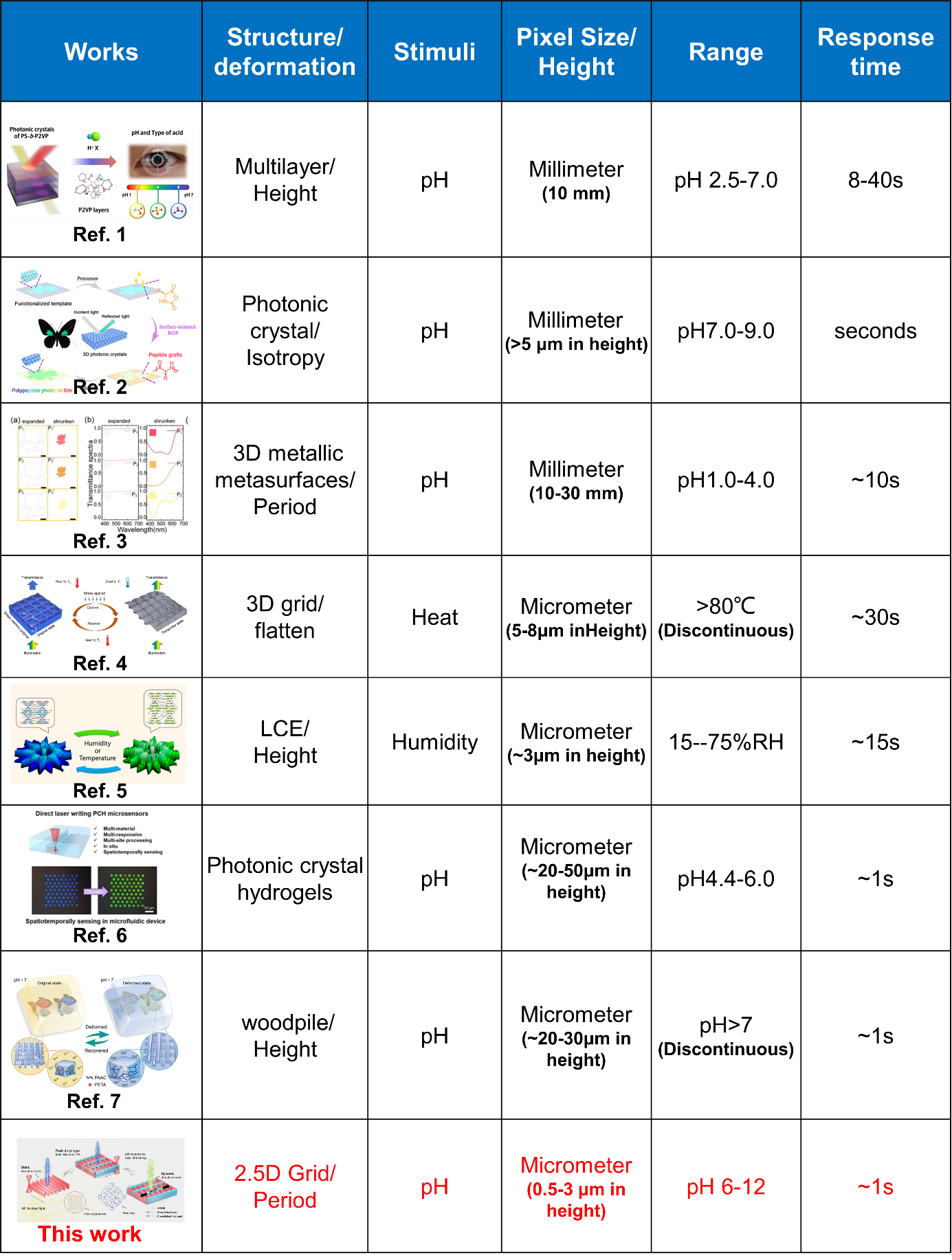
**

**References in Table S1**

1. D. Kim, K.-S. Hwang, J.-H. Kim, C. Lee, J.-Y. Lee, Multituning of structural color by protonation and conjugate bases. ACS Appl. Polym. Mater. **3**(6), 2902-2910 (2021). <https://doi.org/10.1021/acsapm.0c01382>
2. F. Meng, B. Ju, Z. Wang, R. Han, Y. Zhang et al., Bioinspired polypeptide photonic films with tunable structural color. J. Am. Chem. Soc. **144**(17), 7610-7615 (2022). <https://doi.org/10.1021/jacs.2c02894>
3. W. Li, X. Zeng, Y. Dong, Z. Feng, H. Wen et al., Laser nanoprinting of floating three-dimensional plasmonic color in ph-responsive hydrogel. Nanotechnology **33**(6), (2021). <https://doi.org/10.1088/1361-6528/ac345b>
4. W. Zhang, H. Wang, H. Wang, J. Y. E. Chan, H. Liu et al., Structural multi-colour invisible inks with submicron 4d printing of shape memory polymers. Nat. Commun. **12**(1), 112 (2021). <https://doi.org/10.1038/s41467-020-20300-2>
5. M. del Pozo, C. Delaney, C.W.M. Bastiaansen, D. Diamond, A.P.H.J. Schenning et al., Direct laser writing of four-dimensional structural color microactuators using a photonic photoresist. ACS Nano **14**(8), 9832-9839 (2020). <https://doi.org/10.1021/acsnano.0c02481>
6. K. Liu, H. Ding, Z. Chong, Y. Zeng, Y. Niu et al., Direct laser writing photonic crystal hydrogel sensors for in-situ sensing in microfluidic device. Chem. Eng. J. **482**, 148679 (2024). <https://doi.org/https://doi.org/10.1016/j.cej.2024.148679>
7. B. Liu, B. Dong, C. Xin, C. Chen, L. Zhang et al., 4d direct laser writing of submerged structural colors at the microscale. Small **19**(2), 2204630 (2023). <https://doi.org/https://doi.org/10.1002/smll.202204630>

**Supplementary Videos**

**Video S1** Structural colors transitions from water to air

**Video S2** Dynamic structural colors change with pH

**Video S3** Dynamic structural colors under normal incidence and 45° incidence

**Video S4** Dynamic structural colors for information encryption

**Video S5** Dynamic structural colors for visualizing pH diffusion

**Video S6** Integrating dynamic structural colors in microfluidics
